# A systematic review of machine learning applications in predicting opioid associated adverse events

**DOI:** 10.1038/s41746-024-01312-4

**Published:** 2025-01-16

**Authors:** Carlos R. Ramírez Medina, Jose Benitez-Aurioles, David A. Jenkins, Meghna Jani

**Affiliations:** 1 Centre for Epidemiology Versus Arthritis, Centre for Musculoskeletal Research, Division of Musculoskeletal and Dermatological Sciences, The University of Manchester, Manchester, United Kingdom; 2 Division of Informatics, Imaging and Data Science, The University of Manchester, Manchester, United Kingdom; 3 NIHR Manchester Biomedical Research Unit, Manchester University NHS Foundation Trust, Manchester Academic Health Science Centre, Manchester, United Kingdom; 4 Salford Royal Hospital, Northern Care Alliance, Salford, United Kingdom

**Keywords:** Epidemiology, Outcomes research

## Abstract

Machine learning has increasingly been applied to predict opioid-related harms due to its ability to handle complex interactions and generating actionable predictions. This review evaluated the types and quality of ML methods in opioid safety research, identifying 44 studies using supervised ML through searches of Ovid MEDLINE, PubMed and SCOPUS databases. Commonly predicted outcomes included postoperative opioid use (*n* = 15, 34%) opioid overdose (*n* = 8, 18%), opioid use disorder (*n* = 8, 18%) and persistent opioid use (*n* = 5, 11%) with varying definitions. Most studies (96%) originated from North America, with only 7% reporting external validation. Model performance was moderate to strong, but calibration was often missing (41%). Transparent reporting of model development was often incomplete, with key aspects such as calibration, imbalance correction, and handling of missing data absent. Infrequent external validation limited the generalizability of current models. Addressing these aspects is critical for transparency, interpretability, and future implementation of the results.

## Introduction

Opioids, a class of medications used to treat acute and chronic pain, are associated with persistent use, adverse events, unintentional overdoses, and deaths^1^. In 2020, nearly 70,000 opioid-related overdose deaths were reported in the United States^2,3^. Although opioid mortality rates in other countries have not reached these levels, the adverse consequences of prescription opioid use are increasing in countries such as Canada, Australia, and the United Kingdom parallel to increasing prescription use^4–7^ In response to the global impact of harmful opioid use on public health, international efforts have intensified to combat the opioid crisis through the development of effective prevention and treatment strategies^8^.

There has been growing interest in using machine learning (ML) to improve diagnosis, prognosis, and clinical decision-making, a trend largely driven by the widespread availability of large-volume data, such as electronic health records (EHRs) and advances in technology^9^. ML techniques have shown promise in handling large, nonlinear, and high-dimensional datasets and modelling complex clinical scenarios, offering flexibility over traditional statistical models^10^. However, despite their potential, ML applications in healthcare have not consistently led to improved patient outcomes^10^ and often fail to achieve notable clinical impact.^11,12^ The increasing complexity of ML models such as gradient-boosted machines, random forests, and neural networks can reduce their transparency and interpretability, limiting their application within clinical practice.^13–15^ Model development is often driven more by data availability than by clinical relevance^16^, with performance gains not always justifying the trade-off between accuracy and interpretability^17^. Although several ML models have been developed recently to predict opioid-related harms, their clinical utility remains uncertain. We conducted a systematic literature review to identify and summarise available ML prediction models pertaining to opioid-related harm outcomes and assess strengths, limitations, and risk of bias in these studies, providing an overview of the current state-of-the-art ML methods in opioid-drug safety research.

## Results

### Study selection

**Fig. 1:**
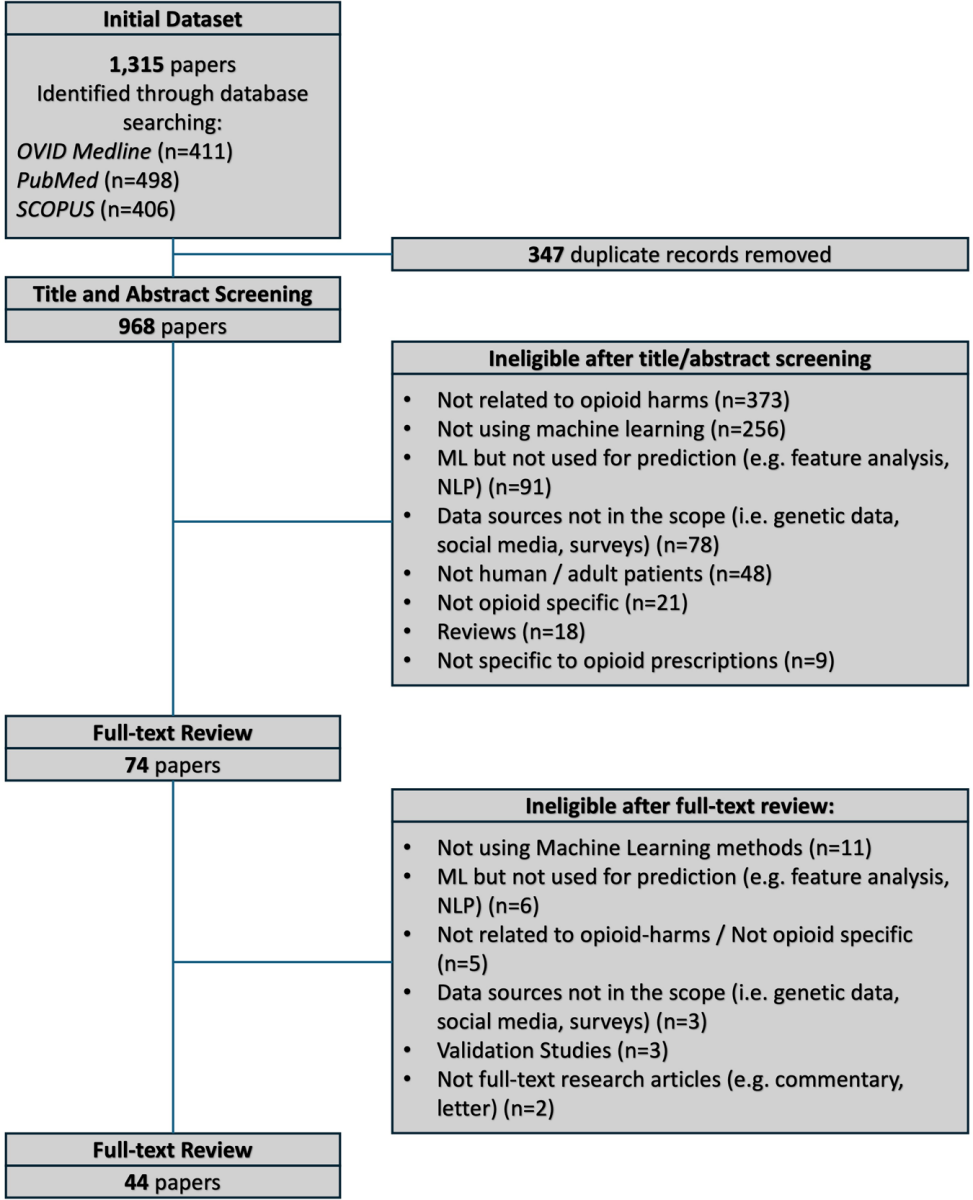
PRISMA flowchart. This flowchart illustrates the inclusion and exclusion of studies at each stage of the systematic review process.

The search yielded 1315 studies (Fig. 1) 347 studies were identified as duplicates, and 894 were excluded after title/abstract screening. Seventy-four full-text articles were retrieved for full review. Among excluded studies, 11 did not use ML methods, six described studies that did not develop predictive models as the main objective, and three relied on data sources other than those in the scope of the review. The flow diagram outlining the study selection process and displaying detailed results of our literature search in accordance with the Preferred Reporting Items for Systematic Reviews and Meta-Analyses (PRISMA) can be found in Fig. 1.

### Overview of included studies

All eligible studies were published between 2017 and 2023, with most of them performed in 2021 (*n* = 11, 25%, Supplementary Table 1). Studies were mainly conducted with data from the United States (*n* = 39,88.6%), with some studies originating in Canada^18–20^ (*n* = 3, 6.8%), Iran (*n* = 1,2.3%)^21^, and Switzerland (*n* = 1,2.3%)^22^. The majority used EHRs as the main data source (*n* = 31, 70%) as opposed to administrative data, with a retrospective cohort study design to perform their analysis. External validation, vital for evaluating the generalisability of the models, was rare and identified for only five studies^23–27^. For two studies this external validation was conducted within the same study of the model creation using a separate dataset^26,27^, and for three models we identified independent studies that aimed to validate the models’ performance in a different setting^23,25^. The performance of the machine learning models assessed in the reviewed studies varied widely, with area under the receiver operating characteristic curve (AUC) values ranging from 0.68^28^ to 0.96^29^ (Table 1). We caution against direct comparison due to differences in training and testing settings across studies. Variations include differences in datasets used, outcomes measured, data partition sizes, and the specific models employed.

**Table 1:** Description of included studies that focussed on machine learning in opioids-associated adverse outcomes

Study	Category	Data	Country	ML Algorithm	Best Performing Model	Results of best performing model	Model Calibration
Behnoush et al. ^21^	Seizure due to acute tramadol poisoning	EHRs	Iran	SVM, NB, ANN, KNN	Logistic Regression	NB (0.71)	Not performed
Anderson et al. ^58^	Postsurgical Opioid Use	EHRs	United States	BBN, RF, GBM, LR	GBM	GBM (AUC. 77, Brier score.10)	Calibration plots & Brier Score
Baxter et al. ^63^	Postsurgical Opioid Use	EHRs	United States	LR, GB, XGBOOST	GBM / Logistic Regression	(0.84)	Not performed
Gabriel et al. ^33^	Postsurgical Opioid Use	EHRs	United States	6 models (LR, RF, SFNN, BRF, BBC, SVM + SMOTE)	RFClassifier	AUC. 94 and. 96 with SMOTE	Brier Score
Giladi et al. ^62^	Postsurgical Opioid Use	EHRs	United States	LR, RF, XGBoost	XGBOOST	XGBOOST (0.72)	Calibration plots & Brier Score
Grazal et al. ^46^	Postsurgical Opioid Use	EHRs & AD	United States	6 models (NB, GBM, EGB, RF, ENPLR, ANN)	Neural Network	AUC. 71, Brier score. 21	Calibration plots & Brier Score
Hur et al. ^28^	Postsurgical Opioid Use	AD	United States	Linear models (SVM) against non-linear (ensemble of decision trees)	XGBoost	Non-linear (AUC. 68) for refills (AUC. 66 for New persistent use)	Not performed
Karhade et al. ^60^	Postsurgical Opioid Use	EHRs	United States	5 models (ENPLR, RF, SGB, NN, SVM)	GBM	AUC. 81, Brier score. 075	Calibration plots & Brier Score
Karhade et al. ^24^	Postsurgical Opioid Use	EHRs	United States	5 models (ENPLR, RF, SGB, NN, SVM)	Neural Network	AUC. 81, Brier Score. 064	Calibration plots & Brier Score
Karhade et al. ^59^	Postsurgical Opioid Use	EHRs	United States	5 models (ENPLR, RF, SGB, NN, SVM)	ENPLR	ENPLR (AUC. 81, Brier Score. 051)	Calibration plots & Brier Score
Karhade et al. ^23^	Postsurgical Opioid Use	EHRs	United States	5 models (RF, SGB, NN, SVM, ENPLR)	ENPLR	ENPLR (AUC. 70, Brier Score. 039)	Calibration plots & Brier Score
Katakam et al. ^32^	Postsurgical Opioid Use	EHRs	United States	5 models (SGB, RF, SVM, NN, ENPLR)	SGB	SGB (AUC. 76, Brier score. 073)	Calibration plots & Brier Score
Klemt et al. ^34^	Postsurgical Opioid Use	EHRs	United States	5 models (ANN, SGB, RF, KNN, ENPLR)	Neural Network	NN (AUC. 87, Brier score. 036)	Calibration plots & Brier Score
Kunze et al. ^45^	Postsurgical Opioid Use	EHRs	United States	5 models (SGB, RF, SVM, NN, ENPLR)	SGB	SGB (AUC. 75, Brier score. 13)	Calibration plots & Brier Score
Lu et al. ^31^	Postsurgical Opioid Use	EHRs	United States	5 models (SVM, RF, XGBoost, AdaBoost, ensemble model)	RF Classifier	AUC. 74, Brier Score. 12	Calibration plots & Brier Score
Zhang et al. ^61^	Postsurgical Opioid Use	AD	United States	7 models (FLR, SLR, LASSO), SVM, two tree-based models (RF and SGB), and tCNN)	Logistic Regression	LR based models (AUC. 835-.847, Sensitivity 74.9–76.5%)	Calibration plots & Brier Score
Bjarnadottir et al. ^50^	Persistent Opioid Use	EHRs	United States	4 models (LR, LASSO, GB, AB)	Logistic Regression	LR (AUC 0.873)	Not performed
Calcaterra et al. ^25^	Persistent Opioid Use	EHRs & AD	United States	(RF, LR, LASSO)	Logistic Regression	LR (AUC 0.68)	Not performed
Held et al. ^22^	Persistent Opioid Use	AD	Switzerland	LR, RF	Logistic Regression	LR (0.927)	Scaled Brier Score
Johnson et al. ^51^	Persistent Opioid Use	AD	United States	6 models (PCA LR, LR, EN, MLP, XGBOOST, XGBOOST validation)	XGBOOST	XGBOOST (0.80)	Evaluated using calibration curves in supplementary file
Mohl et al. ^52^	Persistent Opioid Use	EHRs	United States	4 models (RF, EL, LR, SPVM)	RF Classifier	RF (0.76)	Calibration Plots
Dong et al. ^35^	Overdose	EHRs	United States	RF, DT, LR, DNN, LSTM, ATT	LSTM	LSTM (0.8449)	Not performed
Dong et al. ^36^	Overdose	EHRs	United States	RF, DT, LR, DP	RF Classifier	RF (0.95)	Not performed
Gellad et al. ^37^	Overdose	EHRs	United States	GBM	GBM	GBM (0.86)	Calibration plots & Brier Score
Lo-Ciganic et al. ^38^	Overdose	AD	United States	GBM	GBM	GBM (0.85)	Performed according to the text but further details not provided
Lo-Ciganic et al. ^26^	Overdose	AD	United States	GBM	GBM	GBM (0.85)	Calibration Plots
Lo-Ciganic et al ^39^	Overdose	AD	United States	NN	Neural Network	NN (0.91)	Calibration Plots
Ripperger et al. ^40^	Overdose	EHRs	United States	WL, LASSO, RIDGE, RF	Ridge	RIDGE (0.83)	Calibration plots & Brier Score
Sun et al. ^41^	Overdose	AD	United States	EN, RF, LR	EN	EN (0.887)	Calibration plots & Brier Score
Annis et al. ^42^	Opioid Use Disorder	EHRs	United States	XGBOOST	GBM	0.71	Not performed
Banks et al. ^43^	Opioid Use Disorder	EHRs	United States	LR, RF, DL	RF Classifier	RF (0.79)/RF(0.81)	Not performed
Dong et al. ^44^	Opioid Use Disorder	EHRs	United States	4 models (RG, DT, RF, NN)	Neural Network	NN (0.92) DT (0.88)	Not performed
Gao, W. et al. ^47^	Opioid Use Disorder	AD	United States	5 models (RG, DT, SVM, RF, NN)	Neural Network	NN (0.918) RG (0.915)	Not performed
Kashyap et al. ^48^	Opioid Use Disorder	EHRs	United States	DLM	Deep Learning Models	0.94 ± 0.008	Calibration plots & Brier Score
Liu et al. ^20^	Opioid Use Disorder	EHRs	Canada	Ensemble model	Ensemble method	0.94	Not performed
Lo-Ciganic et al. ^49^	Opioid Use Disorder	AD	United States	(RG, RF, GB, NN)	GBM	GB (0.882) RG(0.880)	Performed according to the text but further details not provided
Segal et al. ^29^	Opioid Use Disorder	AD	United States	GB	GBM	GB (0.959)	Not performed
Che et al. ^55^	Opioid Dependency	EHRs	United States	LR, SVM, RF, DNN	Deep Learning Models	DNN (0.80)	Not performed
Ellis et al. ^56^	Opioid Dependency	EHRs	United States	RF	RF Classifier	RF (Mean 0.863)	Not performed
Fouladvand et al. ^27^	Multiple Adverse Outcomes	AD	United States	LR, RIDGE, LASSO, ELASTIC NET, RF, XGBOOST, DNN	RF Classifier	0.877	Not performed
Sharma et al. ^53^	Multiple Adverse Outcomes	AD	Canada	XGBOOST	XGBOOST	XGBOOST (0.82)	Calibration plot and Brier Score
Sharma et al. ^19^	Multiple Adverse Outcomes	AD	Canada	(XGBOOST, SVM, NB, LR, NN)	Logistic Regression	LR (0.884)	Calibration Plots
Vunikili et al. ^54^	Multiple Adverse Outcomes	EHRs	United States	LR, XGBOOST	XGBOOST	0.83	Not performed
Guo et al. ^57^	Mortality after Overdose	AD	United States	GBM	GBM	GBM (0.71)	Calibration plots & Brier Score

*EHRs* Electronic Health Records, *SGB* Stochastic Gradient Boosting - A machine learning technique that builds an ensemble of decision trees in a sequential manner, where each tree is trained to correct the errors of the previous one, *BBF* Bayesian Belief Network, *RF* Random Forest - An ensemble learning method that constructs a multitude of decision trees at training time and outputs the mode of the classes (classification) or mean prediction (regression) of the individual trees, *SVM* Support Vector Machine - A supervised learning model that analyses data used for classification and regression analysis, known for its effectiveness in high-dimensional space, *NN* Neural Network - A computational model based on the structure and functions of biological neural networks, used for estimating or approximating functions that can depend on many inputs, *ENPLR* Elastic Net Penalized Logistic Regression - A type of linear regression that incorporates penalties from both L1 (Lasso) and L2 (Ridge) regularization methods, used in cases where there are correlated features, *XGBOOST* eXtreme Gradient Boosting - An efficient and scalable implementation of gradient boosting framework by J. Friedman, *GBM* Gradient Boosting Machine - A machine learning technique for regression and classification problems, builds an ensemble of decision trees sequentially, with each tree correcting the errors of its predecessor, *NB* Naive Bayes - A simple probabilistic classifier based on applying Bayes’ theorem with strong (naive) independence assumptions between the features, *LR* Logistic Regression - A statistical model that in its basic form uses a logistic function to model a binary dependent variable, *RIDGE* Ridge Regression - A method of estimating the coefficients of multiple-regression models in scenarios where independent variables are highly correlated, *LASSO* Least Absolute Shrinkage and Selection Operator - A regression analysis method that performs both variable selection and regularization in order to enhance the prediction accuracy and interpretability, *DNN* Deep Neural Network - An artificial neural network with multiple layers between the input and output layers, which can model complex nonlinear relationships, *DT* Decision Tree - A decision support tool that uses a tree-like graph or model of decisions and their possible consequences, *PCA LR* Principal Component Analysis Logistic Regression - A logistic regression model where principal component analysis is used to reduce the dimensionality of the input features before applying the regression, *ANN* Artificial Neural Network - A computing system vaguely inspired by the biological neural networks that constitute animal brains, consisting of interconnected nodes that process data and learn patterns, *KNN* K-Nearest Neighbours - A non-parametric method used for classification and regression, where the input consists of the k closest training examples in the feature space, *ADABOOST* Adaptive Boosting – An ensemble method that combines multiple weak learners to create a strong classifier by focusing on correcting errors from previous models, *SMOTE* Synthetic Minority Over-sampling Technique addresses class imbalance in machine learning by generating synthetic data points for the minority class. This technique helps balance class distribution.

The reliability of risk predictions (calibration metrics) was not reported for a notable portion of the reviewed studies (*n* = 18, 40.9%). These metrics are essential for evaluating how well the predicted probabilities align with the actual observed outcomes [Table 1].

### Outcomes of interest of the reviewed studies

Within clinical prediction, diagnostic prediction models are used to estimate the probability of a disease that is already present, while prognostic models aim to assess the risk of future health conditions^30^. The largest category of studies in this review focused on prognostic models for postoperative opioid use, with 15 studies (34%)^23,31–44^. The majority of these studies examined hip or knee arthroscopy^31,45,46^ and spine surgery^32–34^. Other primary outcomes of prediction models included opioid overdose prediction (*n* = 8, 18%)^26,35–41^, opioid use disorder (*n* = 8, 18%)^20,29,42–44,47–49^ and prolonged opioid use (with varying definitions, to be detailed in subsequent sections) (n = 5, 11%)^22,25,50–52^. Additionally, four studies (7%) utilised a composite outcome that included hospitalisations, emergency department visits, substance abuse and mortality^19,27,53,54^. A smaller subset of studies concentrated on other opioid-related harms as their main outcomes. Specifically, two studies (5%) focused on opioid dependence^55,56^, one (*n* = 1, 2%) on mortality^57^, and one on seizure after tramadol overdose (*n* = 1, 2%)^21^.

In the following sections, we provide a detailed explanation of each identified category and an overall summary of the methods, data collection procedures, and statistical analyses used in these studies to examine their research questions. Full summary of included studies, predictor variables (Supplementary Table 2) and numbers of participants/ outcomes (Supplementary Table 3) are included in the online supplementary information.

### Prognostic models to predict opioid use after surgery

Fifteen studies (34%)^23,24,28,31–34^^,45,46,58–63^ were identified with the primary objective of developing a prognostic predictive model using machine learning to address postoperative opioid use.

All eligible studies were conducted within the last four years, with most of the studies developed within 2022 (*n* = 4/15, 27%)^31,33,34,46^, while the earliest studies were from 2019 (*n* = 3/15, 20%)^24^^,59,60^. The studies included in this systematic review used various terms to describe their outcomes related to postoperative opioid use, such as “prolonged,” “chronic use”, “persistent,” “sustained,” and “extended” opioid use. In the absence of a universally agreed definition that can yield different prevalence results^64^, these definitions varied and encompassing different metrics at various time points. Examples include any opioid prescription filled between 90 to 365 days after surgery, continued opioid use beyond a 3-month postoperative period, use extending up to 6 months, continued postoperative opioid use at specific intervals (14 to 20 days, 6 weeks, 3 months, 6 months), filling at least one opioid prescription more than 90 days after surgery, uninterrupted filling of opioid prescriptions for at least 90 to 180 days, and opioid consumption continuing for at least 150 days following surgery.

Datasets and Sampling: Thirteen studies (*n* = 13/15, 87%) used EHRs as the main data source (two from a military data repository), and the remaining used insurance claims (*n* = 2/15, 13%). All the prediction models in these studies were developed with data from patients in the United States, and external validation could only be identified for two of the developed prediction models^23,24^ (both in Taiwanese cohorts)^65,66^. External validation remains to be performed in non-US and non-Taiwanese patient groups for all these developed models.

Sample sizes across the included studies exhibited substantial variation, ranging from 381^31^ to 112,898 patients^28^ (mean = 13,209.6, median= 5,507). The overall number of outcome events was considerably smaller for most of the studies, with the percentage of outcome incidence ranging from 4%^23^ to 41%^46^ (mean = 13%, median= 10%). Although data to develop the prediction models was imbalanced (with an outcome frequency of less than 20%) for thirteen studies in this group, only eight studies explicitly acknowledged it and addressed using techniques such as oversampling (*n* = 2/15, 13%)^31^^,^^33^ or by reporting the area under the precision-recall curve (AUPRC) (*n* = 5, 33%)^23,60^. AUPRC provides a more informative measure of classifier performance on imbalanced data sets than more common classification metrics such as AUC and accuracy, which can be misleading in such a scenario. Many of these studies only used demographic and preoperative predictors to build their predictive models. (*n* = 8/15, 53%).

When addressing missing data, only eight studies explained how they were handled. Most of them used multiple imputation method with ‘missForest’ (*n* = 7/15, 46%), a popular Random Forest (RF)-based missing data imputation package in software. One used the Multivariate Imputation by Chained Equations (MICE) package in R. While only two studies explicitly mentioned how continuous variables were handled^46,58^, eight suggested that they were variables were kept as continuous in downstream analysis; for five studies this was left unclear (33%).

Most studies (*n* = 8/15, 53%) that developed prognostic models to predict opioid use after surgery used a group of five algorithms for predictive modelling due to their ability to handle complex data and produce accurate predictions: Stochastic Gradient Boosting, RF, Support Vector Machine, Neural Network, Elastic-net Penalised Logistic Regression. Other studies also incorporated XGBoost (*n* = 4 out of 15, 27%) and LASSO (*n* = 4 out of 15, 27%) algorithms in their analyses. The most common algorithms were RF (*n* = 12/15, 80%) and Elastic-net penalised logistic regression (*n* = 9/15, 60%). Values reported for the area under the receiver operating characteristic curve (AUC) ranged from 0.68^28^ to 0.94^33^. Notably, logistic regression, along with its regularized form, Elastic Net, were consistently reported to perform on par to the performance ensemble methods such as random forests and gradient boosting machine algorithms (*n* = 7/15, 46%). Calibration metrics were reported for most of the studies (*n* = 13/15, 87%), which included calibration plots, intercept, slope and Brier Score (ranging from 0.037 to 0.136) for these studies.

Patient subtypes: Most of the research on opioid use after orthopaedic surgery included patients who underwent a specific single surgical procedure, mainly arthroplasty (*n* *=* 3/15, 20%)^31^^,^^45^^,^^46^ and spine patients (*n* = 3/15, 20%)^23,24,61^.

### Prognostic models to predict opioid use disorder

Eight studies were identified^20,29,42–44,47–49^. All, except for one Canadian study^20^ used data from the United States. The sample size varied significantly, ranging from 130,120^29^ to 5,183,566 ^44^ (mean= 1,116,761, median= 361,527) with the percentage of outcome events ranging from 1% to 4% (Mean = 2%, Median=2%). Data in this category of studies were highly imbalanced. Two studies used oversampling techniques to handle classification imbalance^20,42^ and three more reported AUPRC^44,47,48^. No external validation was identified for any of these models. Data-driven variable selection was only mentioned in two studies, using the Andersen Behavioural Model^42^ and LASSO logistic regression methods alongside to supervised machine learning algorithms^44^. In terms of performance, Gradient Boosting and neural network classifiers had the best performance based on AUC ranging from 0.88^49^ to 0.959^29^ with no overall performance benefit over logistic regression. In contrast, Support Vector Machine (SVM) was the worst-performing algorithm^47^. Very few studies (*n* = 2/8, 25%) reported performing calibration as part of their analysis but only one study appropriately reported it, including calibration plots, intercept, slope, and Brier Score^49^.

### Prognostic models to predict opioid overdose

Eight studies were identified^26,35–41^. All the studies in this subgroup were conducted in the United States, four (*n* = 4 out of 8, 50%) used EHR and four used administrative and insurance claims data from sources like Medicaid or Medicare (*n* = 4 out of 8, 50%), with the majority being developed in 2021 (*n* = 3 out of 8, 38%). The population sample size in these studies ranged from 237,259 to 7,284,389, with the percentage of outcome events ranging from 0.04%^37^ to 1.36%^26^. Although data imbalance was only directly addressed with imbalance data techniques for three studies^36,37,40^ it was acknowledged by all studies and additional performance metrics were reported, including AUPRC^26^^,38,39^. Only one study was externally validated^26^. The models developed in this category had high c-statistics, ranging from 0.85^26^ to 0.95^35^ and good prediction performance.

### Prediction models for persistent opioid use

Five studies were identified.^22^^,25,50–52^ This included three with an explicit chronic opioid use outcome, one focusing on long-term opioid use prediction and one predicting progression from acute to chronic opioid use^51^. All of them were retrospective studies, with the oldest dating back to 2018^25^. The population sample size ranged from 27,705 to 418,564 patients, with the percentage of outcome events ranging from 5%^25^ to 30%^51^. Only one study addressed class imbalance but used a down-sampling approach^25^, which risks discarding valuable information potentially leading to reduced model accuracy. All studies had internal validation, but only one was externally validated using data from two additional healthcare organizations^25^ (Supplementary Table 4). The top-performing algorithms in terms of AUC were logistic regression (3 out of 5, 60%)^25^, RF classifier (*n* = 1 out of 5, 20%)^52^, and XGBoost (*n* = 1 out of 5, 20%)^51^, with SVM being the worst performing (AUC = 0.72)^51^. One study also found that class balancing did not have a significant impact on model performance for most models, despite the relatively rare outcome^50^. Only one study in this category (*n* = 1/5, 20%) reported and provided details of calibration for their analysed models^52^. Calibration details reported across studies are presented in Supplementary Table 5.

### Prediction models for opioid dependence

Two studies were identified^55,56^. The sample size ranged from 102,166 to 199,273. The percentage of outcome events ranged from 0.7%^55^ to 3.9%^56^. While both studies used large samples from US EHRs, Che et al benefited from having data from multiple hospitals. Additionally, Che et al. had access to a clear diagnosis of “opioid dependence” in the patient records^55^ whilst Ellis et al. had to rely on various forms of substance dependence for the definition of their outcome (not exclusive to opioids)^56^. Both studies addressed the small number of outcomes with class imbalance techniques and showed a good discrimination performance of 0.8^55^ and 0.87^56^. Che et al. reported that deep learning solutions achieved superior classification results and outperformed other baseline methods. None of the studies reported calibration measures nor were found to be externally validated.

### Prognostic models to predict other opioid-related harms

Only a limited number of studies investigated opioid-related harms beyond those commonly addressed above. Among these studies, four developed prognostic models using administrative health data sets to predict hospitalization, emergency visits, and mortality^19,27,53,54^. For one of these studies, simpler linear models carried higher discrimination and performance^19^ and for another, although the final model using XGBOOST had high discrimination performance, the calibration plot showed a consistent overestimation of risk^53^. Vunikili et al. used a retrospective cohort of patients to build a model predicting opioid abuse and mortality. The XGBoost algorithm outperformed logistic regression for classifying patients susceptible to “opioid risks”^54^. Fouladvand, et al. focused on predicting whether a person experienced opioid abuse, dependence, or an overdose event (opioid-related adverse outcomes) during the 6-month period after surgery. The best predictive performance was achieved by the RF model, with an AUC of 0.87. The model was well calibrated and had good discrimination and was externally validated using data from other states within the United States^27^. Other studies in this category developed models to predict mortality risk after nonfatal opioid overdose (using gradient boosting machines)^57^ and predict seizure due to tramadol poisoning (using machine learning models that did not show significant performance improvements compared to the logistic regression model)^21^.

### Risks of bias

In accordance with the Prediction Model Risk of Bias Assessment Tool (PROBAST) guidance, we systematically evaluated the risk of bias in 44 identified studies across four key domains: participants, predictors, outcomes, and analysis (Table 2). We found that 16/44 studies had a high risk of bias in at least one domain and 19/44 unclear in at least one domain due to lack of information. Only 9/44 studies were found to have a low risk of bias across all domains. A summary of the risk of bias assessment for machine learning algorithms grouped by type of outcome is presented in Supplementary Figs. 1–4.

**Table 2:**
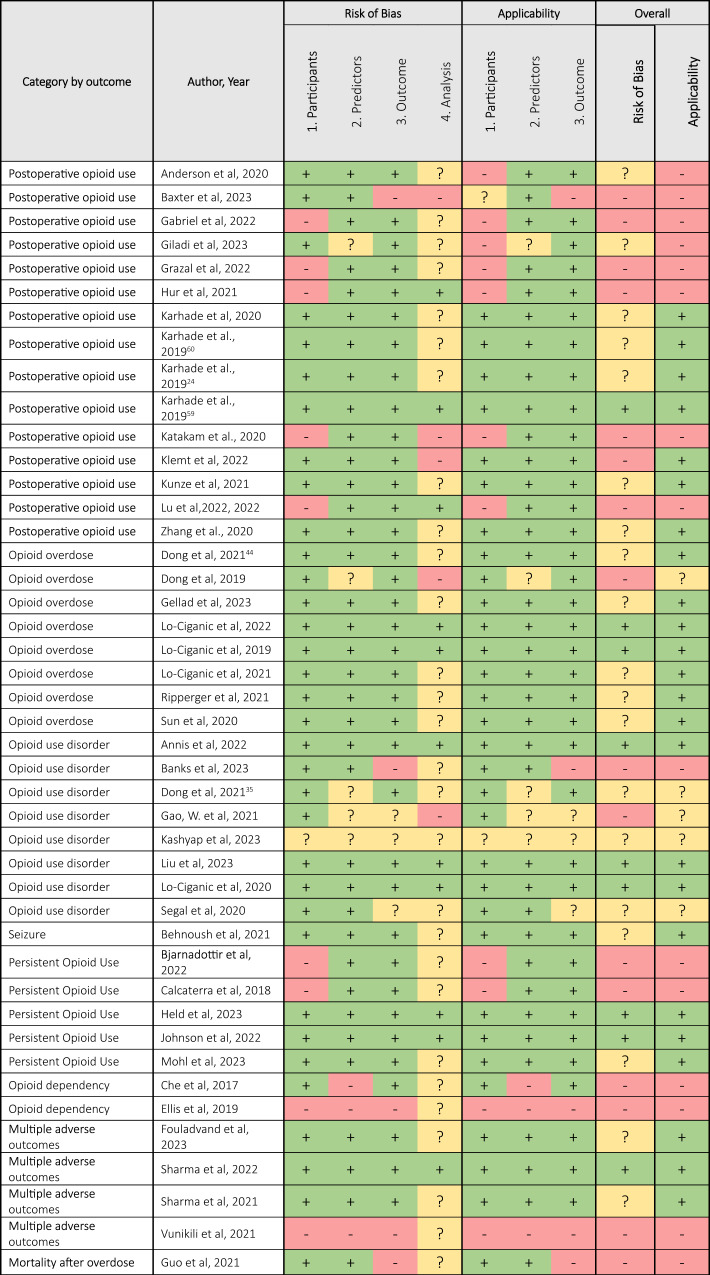
PROBAST results for each domain considered for each paper included in the systematic review

Risk of bias and applicability were assessed for each study using the Prediction model study Risk of Bias Assessment (PROBAST) tool. The assessment involves scoring each domain as having low (+), high (-) or unclear risk of bias (?) based on predefined criteria^23^.

Participants: Ten studies had a high (9/44) or unclear (1/44) risk of bias for their participants. This was primarily due to the following issues: (1) the model was built using datasets from a single health centre (2) the paper’s inclusion and exclusion criteria is not described with enough details to be reproducible and/or (3) there are large differences in demographics of the target population and the population the data on which the model was built upon.

Predictors: Among the studies, 5/44 lacked sufficient information on predictors, rendering bias assessment challenging. For 36 studies, the risk of bias was deemed low due to the comprehensive description of predictor definition, selection, assessment, and handling during analysis. Conversely, 3/44 studies exhibited a high risk of bias, primarily due to insufficient details for the reproducibility of the analysis.

Outcomes: In terms of outcome variables, 36/44 studies demonstrated low risk, 3/44 were unclear, and 5/44 exhibited a high risk of bias. The key contributing factor was the inconsistency between the studies’ stated objective of predicting opioid-related harms and the inclusion of outcomes not specific to opioid-related harms.

Analysis: Only 11/44 studies were evaluated to have a low risk of bias in their analysis, either by adhering to TRIPOD guidelines, following the Guidelines for Developing and Reporting Machine Learning Models in Biomedical research, or providing sufficient detail on the analysis methodology. Conversely, 28 studies lacked adequate information, leading to classification as unclear risk, while 5 studies exhibited a high risk of bias. Common shortcomings included inadequate information for reproducibility, such as missing data handling, class imbalance handling, hyperparameter description, tool/library versions, and non-availability of code (Table 3).

**Table 3:** Description of Studies describing class imbalance considerations hyperparameters and data/ code availability

Author	Year	Class imbalance considered	Hyperparameters described	Version of tools and libraries used	Dataset available	Code available
Anderson et al.	2020	Not mentioned	Yes	Partial	No	No
Annis et al.	2022	SMOTE and oversampling	Yes	Yes	No	No
Banks et al.	2023	Not mentioned	Yes	Yes	On request	On request
Baxter et al.	2023	Not mentioned	Not mentioned	Partial	No	Unclear
Behnoush et al.	2021	Not an imbalanced outcome	Not mentioned	Partial	No	No
Bjarnadottir et al.	2022	Stratified bootstrapping	Yes	Yes	No	On request
Calcaterra et al.	2018	Down-sampling	Not mentioned	Not mentioned	No	No
Che et al.	2017	Down-sampling	Not mentioned	Partial	No	No
Dong et al.^44^	2021	Not mentioned	Not mentioned, but hyper-parameter tuning suggested	Yes	No	Yes
Dong et al.^35^	2021	Not balanced	Not mentioned	Not mentioned	No	Yes
Dong et al.	2019	Under sampling	Not mentioned	Yes	No	No
Ellis et al.	2019	Over sampling	Partial	Partial	No	No
Fouladvand et al.	2023	Yes	Yes	Not mentioned	No	No
Gabriel et al.	2022	Oversampling	Not mentioned	Yes	On request	Unclear
Gao, W. et al.	2021	Not mentioned	Not mentioned	Not mentioned	No	No
Gellad et al.	2023	Down-sampling	Partial	Partial	No	No
Giladi et al.	2023	Not mentioned	Yes	Yes	No	No
Grazal et al.	2022	Not mentioned	Not mentioned	Partial	No	Yes
Guo et al.	2021	Not mentioned	Yes	Yes	No	No
Held et al.	2023	To control imbalance a second RF model was fitted on stratified random samples.	Not mentioned	Yes	On request	On request
Hur et al.	2021	Not mentioned	Yes	Yes	No	Yes
Johnson et al.	2022	Not mentioned but AUPRC provided	Yes	Not mentioned	No	Yes
Karhade et al.	2020	Not mentioned	Not mentioned	Partial	No	Mentioned but not accessible (an error occurs).
Karhade et al.,^60^	2019	Not mentioned	Not mentioned	Partial	No	No
Karhade et al.,^24^	2019	Not mentioned	Not mentioned	Partial	No	No
Karhade et al.,^59^	2019	Not mentioned	Not mentioned	Partial	No	No
Kashyap et al	2023	Not mentioned	Not mentioned	Partial	No	No
Katakam et al.	2020	Not mentioned	Not mentioned	Partial	No	No
Klemt et al.	2022	Not mentioned	Not mentioned	Partial	No	No
Kunze et al.	2021	Not mentioned	Not mentioned	Partial	No	No
Liu et al.	2023	Yes	Yes	Partial	No	No
Lo-Ciganic et al.	2021	Not mentioned, but AUPRC reported	Not mentioned	Not mentioned	On request	On request
Lo-Ciganic et al.,	2022	Not mentioned, but they used different measure to evaluate model performance.	Not mentioned, but hyper-parameter tuning suggested	Partial	No	No
Lo-Ciganic et al.,	2020	Not mentioned	Not mentioned, but hyper-parameter tuning suggested	Not mentioned	On request	No
Lo-Ciganic et al.,	2019	Not mentioned, but AUPRC reported	Not mentioned	Partial	No	No
Lu et al.	2022	Yes	Not mentioned	Partial	No	No
Mohl et al	2023	Yes	Not mentioned	Not mentioned	No	No
Ripperger et al.	2021	Under-sampling	Not mentioned	Not mentioned	No	No
Segal et al.	2020	Not mentioned	Yes	Partial	Yes	No
Sharma et al.	2022	Weight scaling	Yes	Yes	No	No
Sharma et al.	2021	Not mentioned	Not mentioned	Yes	No	No
Sun et al.	2020	Not mentioned	Not mentioned, but hyper-parameter tuning suggested	Yes	No	No
Vunikili et al.	2021	Oversampling	Not mentioned	Not mentioned	No	No
Zhang et al.	2020	Not mentioned	Not mentioned	Not mentioned	No	Mentioned but not accessible (an error occurs).

*AUPRC* area under the precision-recall curve, *SMOTE* Synthetic Minority Over-sampling Technique.

Public availability of the algorithms and models: Only 5/44 studies published the code for reproducing their results, 4/44 suggested that it is available on request (Table 3).

## Discussion

This systematic review summarises the extensive efforts of the international community to address the opioid crisis by developing ML prediction models. Comparing complex models with more interpretable ones is necessary to assess whether the trade-offs between performance and interpretability is justified^17^. Studies in this area have primarily been published in recent years and show promise and potential in identifying patients at risk of opioid-related harms in North America (*n* = 36/44, including studies from the US *n* = 33/44 and Canada *n* = 3/44). However, our findings reveal specific methodological limitations, including poor transparency, inadequate machine learning methodology disclosure, limited reproducibility, and biases in study design. The existing literature often relies on C-statistics alone as a performance metric, which may lead to overestimating the advantages of the prediction tools for rare outcomes. In a highly imbalanced dataset, as can be the case with opioid-adverse events, the ROC curve can be overly optimistic. ROC-AUC performance metric is calculated based on the true positive rate and false positive rate^67^. The true positive rate (also known as sensitivity or recall) represents the proportion of actual positives correctly identified as such. The false positive rate represents the proportion of actual negatives incorrectly identified as positives^68^. When the true negative rate is much larger (also known as the specificity or calculated as 1- false positive rate), even a large number of false positives might result in a low false positive rate, artificially inflating the AUC and giving a false impression of high performance (AUC close to 1) even when the model is not effectively identifying the few positive cases. Considering additional metrics, such as the Precision-Recall curve, F1 Score, or other metrics could better reflect model performance in imbalanced scenarios^69^. The absence of calibration reporting in a substantial proportion of the examined studies (41%) raised concerns about the reliability of the reviewed prediction models. Details about internal and external validation for each of the studies is included in Supplementary Table 4.

Our review suggests that developed ML models for predicting opioid related harms primarily showcase ML’s potential rather than being created for clinical application, making them restricted for research purposes only. Key barriers to the practical application of these prediction models include the lack of external validation, accessibility, and the infrastructure to process risk scores and to ensure the algorithms usability and effectiveness^26^. Given the importance of model interpretability for government policymaking, we suggest using explanatory algorithms such as Local Interpretable Model-Agnostic Explanations (LIME)^70^ and Shapley Additive Explanations (SHAP)^71^. Explanatory algorithms could allow clinicians and patients to understand the relationships between the variables in the model, making them more transparent and interpretable. In the reviewed studies only 36% (*n* = 16/44) included them in their analysis. However, these methods have limitations. Both LIME and SHAP provide insights based on the correlations defined by the model, but they do not offer causal explanations. Additionally, these methods may struggle with collinearity; for example, in the presence of highly correlated variables, SHAP values might be high for one variable and zero or very low for another^72^. Furthermore, calculating SHAP values can be computationally expensive, especially for large datasets or complex models. Lastly, the SHAP method assumes the model is a “black box”, meaning it does not incorporate information about the model’s internal structure^73^.

Only five models reached the threshold of methodological quality, reproducibility, and external validation that is required to support utilization in clinical practice. For most studies (*n* = 39,89%) there was an absence of reporting on how results could be implemented clinically or for external validation. This limitation emphasises why deployment of ML models in real-world clinical settings remains uncommon^11^. The generalisability of the reviewed models is also limited, due to the use of data from limited sources such as being from a single centre,^21^^,62,63^ a single geographic area,^26^ or sources that don’t fully reflect the population they aim to study^58^.

Sampling bias in data collection was found to be a common problem in studies using insurance claims datasets (which don’t fully capture the demographics most at risk for opioid-related harms) and research based on military personnel data (which may focus on a younger, predominantly male population^58^). Some studies (*n* = 4,9%) also revealed risk of racial bias and found differences in the racial composition of the groups used to develop the model versus those used to test it^62^, which could particularly impact under-represented subgroups of patients and are important considerations in the development of ML algorithms^74^.

Predictor variables varied significantly across the studies that developed clinical prediction models, with various studies not providing a complete list of variables used, which raises concerns about reproducibility. While demographic factors such as age and sex were commonly reported, only 15 out of 44 studies (*n* = 34%) explicitly included socioeconomic status as a predictor in their models. The lack of consistent reporting and inclusion of variables such as socioeconomic status in many studies may weaken the robustness of the ML models and limit their generalisability.

For the identified models aimed to predict mortality, their accuracy is questionable since the cause of death was not always available and may not necessarily be related to opioid exposure^57^.

For the selection of model predictors, authors chose them based on theory or previous literature. There is an opportunity to explore the benefits of a pure data mining approach and compare its utility against with models developed with user input^43^. The addition of opioid dosage (e.g. as morphine milligram equivalents per day) in future prediction models aimed for clinical implementation is crucial. Even though opioid dose has been found to be associated with a higher likelihood of opioid-harms by several studies^43,75^, less than half of the reviewed studies (*n* = 21,44%) considered opioid dosage, often due to lack of data availability. Additionally, considering the time-varying nature of opioid use with discrete periods of being on or off the drug, transparent preparation of this type of data is especially important to avoid misclassification of opioid exposure that can considerably affect the point estimates associated with adverse outcomes^76^. None of the reviewed models that predicted adverse opioid outcomes employed ML for time-to-event analysis, indicating a potential area for future development.

Recent advancements in deep learning have shown strong predictive capabilities of transformer-based models, which could improve the performance of existing ML models in fields like healthcare^77^. Originally developed for natural language processing, their ability to capture structure in human language could generalise to life-sequences, such as socio-economic and health data, for classification tasks. However, despite their ability to provide highly accurate predictions and often outperform state-of-the-art algorithms, transformer-based models are still nascent in the context of clinical prediction modelling and present several current challenges. These include their highly complex architectures with multiple layers of attention mechanisms, a vast number of parameters, high computational demands, domain-specific adaptations and the need for large, high-quality, well-curated datasets^78^.

We would like to acknowledge strengths and limitations of the current study. This review evaluated and summarised systematically the various applications of machine learning models in addressing prescription opioid-related harms in adults. We acknowledge that some authors may have performed model calibration as well as handling class imbalance and missing data but not mentioned so in their studies. Therefore, we labelled risk of bias as “unclear” to avoid overestimating problems. In cases with not enough information on model development, performance, and calibration, it is hard to interpret the validity of the results. This study complements the works by Garbin et al. and Emam et al., by focusing specifically on machine learning-based prognostic models that predict opioid-related harms and assessing bias risk using PROBAST. Systematic reviews have been partially conducted addressing the opioid epidemic in the present year^79,80^, mainly focusing on postoperative opioid misuse, while other opioid-related harms have not undergone similar assessments. In addition, we report on specific areas of development that could be useful for future research building on the work done in this field thus far.

The application of machine learning in predicting opioid-related harms and identifying at-risk patients has shown promising results, offering valuable insights into the complex landscape of opioid use. Currently, most of these models have not been implemented in clinical practice. Instead, they serve as research tools, illustrating the potential power of machine learning in enhancing our understanding of opioid-related harms. One of the key limitations of the current literature is the lack of transparency reporting and limited external validation studies for the developed prediction models using ML. In navigating the future integration of ML in opioid-related harm prediction, researchers should prioritise comprehensive validation efforts, ensuring that these models are robust, generalisable, and properly reported to be reproducible.

## Methods

### Identification of studies

To identify relevant articles that were published from the inception of records until the 12th of October 2023, an all-time search was conducted using Ovid MEDLINE, PubMed, and SCOPUS databases. The search was performed without any restrictions on publication date, language, or study design to ensure all relevant studies were included. To improve transparency, we followed the Preferred Reporting Items for Systematic Reviews and Meta-Analyses (PRISMA) checklist^81^. Using the Population, Intervention, Comparison, and Outcome (PICO) framework, we defined our relevant search: different types of prescription opioids (population), a broad definition of ML (intervention) and opioid-related harms (outcome). We used a combination of MeSH terms, keywords, and Boolean operators to construct our search string (full search details are provided in Supplementary Table 6).

### Inclusion criteria

We used a three-stage process to identify the studies to be included in this review (Fig. 1). Articles were eligible for full-text review if they detailed construction of one or more machine learning-based prediction models, focusing on a primary outcome directly associated with prescription opioid-harms. We specifically sought studies addressing harms such as dependence, misuse, abuse, overdose, hospitalisations, and death. It is important to note that the terminology used to describe these harms varied across studies. Terms such as chronic opioid use, long-term opioid use, and persistent opioid use may have been used interchangeably or with different definitions^64^. Opioid dependence, whilst sometimes used synonymously in the literature, is the adaptation to repeated exposure to some drugs and medicines usually characterised by tolerance and/or withdrawal and may be inevitable for those on long-term opioids^82^. Addiction relates to dependence with a compulsive preoccupation to seek and take an opioid despite consequences^82^. To ensure a comprehensive coverage of opioid-related harms, we included studies that used a range of these terms and their synonyms (Supplementary Table 6). Regarding methodology, we considered a study to use supervised machine learning if it reported any statistical learning technique to predict outcomes of interest or categorise cases based on a known ground truth, regardless of the terminology used by the authors^83,84^. We excluded studies that developed models based solely on regression techniques. In addition, eligible studies had to predominantly utilise data from EHRs or patient administrative records.

### Exclusion criteria

We excluded studies from our review if they met any of the following criteria: 1) studies that primarily focused on risk factor identification without a clear emphasis on predictive modelling; 2) studies utilising ML to process and analyse human language, enhance the reading of images, to understand user-generated text; 3) studies employing genetic traits or molecular markers as predictive factors; 4) studies that were systematic reviews, letters, conference abstracts or commentaries: 5) studies relying on data sources other than EHRs or administrative patient data (e.g. public domain data such as social media, internet searches, and surveys); 6) studies not concentrating on human subjects or those specifically targeting paediatric populations (patients younger than 18 years old). Full inclusion and exclusion criteria can be found in [Table 4].

**Table 4 -:** Eligibility criteria of included studies

Parameter	Inclusion Criteria	Exclusion Criteria
General	Full-text articles published in any language from inception to October 12th, 2023	Abstracts, Reviews, Letters
Participants	Adult human patients	Paediatric population Non-human subjects Patient data from public domain data sets such as social media and surveys
Intervention	NA	NA
Comparator	ML Algorithms used, performance metrics such as AUPRC, AUC, Accuracy, Precision, Sensibility, Calibration, Brier Score, F1 Score.	
Outcome	Prediction	- Studies that applied natural language processing (NLP) to understand user-generated text for pharmacovigilance - Validation studies
Study Design	Cohort studies	Commentaries and opinion pieces without primary data

*AUC* area under the curve, *AUPRC* area under the precision-recall curve, *ML* machine learning

### Screening process used to select studies for inclusion

All abstracts were screened by C.R. with uncertainties being resolved by consensus with M.J. The full text of selected abstracts was assessed for eligibility by C.R., with the supervision of M.J.

### Data extraction, quality assessment and analysis

In our data extraction using the “CHecklist for critical Appraisal and data extraction for systematic Reviews of prediction Modelling Studies” (CHARMS) checklist Bias assessment process was performed using the Prediction model Risk of Bias Assessment (PROBAST) tool. We followed the standardised checklist designed by B.M. Fernandez-Felix, et al.^85^. The data collected for each study included information such as study design, sample size, number of outcome events, main data sources, machine learning algorithms used, the best and worst algorithms based on C-statistic, outcomes measured and their definition, mention of class imbalance (imbalance between the frequency of outcome events and nonevents)^86^ and missingness, calibration measures reported and details of internal and external validation. The full list of extraction items can be found in the Supplementary Table 6. To identify external validation studies an all-time search was conducted on October 12, 2023, using Ovid MEDLINE, PubMed, and SCOPUS databases (full search details are provided in Supplementary Table 6).

## Supplementary information

Supplementary Material

## Data Availability

All data generated or analysed during this study are included in this published article and its supplementary information. All study materials are available from the corresponding author upon reasonable request.
