# Supplementary material for: A systematic review of machine learning applications in predicting opioid associated adverse events
Source: NPJ Digit Med. 2025 Jan 16;8:30. doi: 10.1038/s41746-024-01312-4 (PMC11739375; doi:10.1038/s41746-024-01312-4)
Supplement: Supplementary file 1 — Supplementary Material [file 41746_2024_1312_MOESM1_ESM.pdf]

# Supplementary Material

## Supplementary Table 1: Number of studies by year

| Year | Number of Studies |
|---|---|
| 2017 | 1 |
| 2018 | 1 |
| 2019 | 6 |
| 2020 | 7 |
| 2021 | 11 |
| 2022 | 9 |
| 2023 | 9 |
| **Grand Total** | **44** |

## Supplementary Table 2: Predictor variables reported by the studies

| Category | Author | Year | Predictors included |
|---|---|---|---|
| **Seizure** | Behnoush et al | 2021 | **Demographic** variables included age, sex, ingested dose, manner of poisoning, history of addiction, and the interval between consumption and seizure.<br>**Clinical variables** encompass systolic and diastolic blood pressure, Glasgow Coma Scale (GCS) score, pulse rate (PR), and respiratory rate (RR).<br>**Paraclinical variables** involve arterial blood oxygen pressure (PO2), partial pressure of carbon dioxide (PCO2), blood oxygen saturation (O2 sat), blood bicarbonate level, platelet count, haemoglobin level (Hb), white blood cell count (WBC), blood sugar, and blood sodium and potassium levels. |
| **Postsurgical** | Anderson et al | 2020 | **Demographic** variables (age, gender, and self-reported patient race)<br>**Military employment characteristics** (rank, service, total time deployed, and rank)<br>**Pharmacy data** (quantity of opioids prescribed at each time period in morphine equivalents, number of refills, and the clinic from which patients received prescriptions). |
| **Postsurgical** | Baxter et al | 2023 | **Demographic** variables, including sex race, ethnicity, comorbidities, and prior exposure to opioids. |
| **Postsurgical** | Gabriel et al | 2022 | **Demographic, Preoperative, intraoperative and postoperative.** Specifically, covariates included in the models were surgical procedure (total hip arthroplasty (posterolateral approach vs. anterior approach), total knee arthroplasty, revision total hip arthroplasty, and revision total knee arthroplasty), age, sex, body mass index, English as a primary language, preoperative opioid use, previous joint replacement surgery, osteoarthritis severity in the operative limb, hypertension, coronary artery disease, chronic obstructive pulmonary disease, asthma, obstructive sleep apnoea, diabetes mellitus (non-insulin vs insulin-dependence), psychiatric history (anxiety and/or depression), active alcohol history (defined as ≥2 drinks per day), active smoking history, active marijuana use, use of perioperative regional nerve block, primary anaesthesia type (neuraxial vs general anaesthesia), intraoperative ketamine use (yes or no), opioid use on postoperative day 1 (measured in intravenous morphine equivalents (MEQ)), amount of prescription opioids given at discharge (MEQs), and hospital length of stay (days) |
| **Postsurgical** | Giladi et al | 2023 | **Demographic data, electronic health record (EHR) details, questionnaire scores, self-reported medical conditions, and socioeconomic status.** Key demographic variables include age, gender, ethnicity, race, body mass index (BMI), and smoking status.<br>**Clinical information** is provided by variables such as surgery type (categorized into bone vs. soft tissue and trauma-related vs. elective), opioid use disorder, drug use disorder, alcohol use disorder, cancer, post-traumatic stress disorder (PTSD), and other self-reported medical conditions such as anaemia, asthma, diabetes, and heart disease. The study also considers past and current use of medications, including opioid receptor activators and blockers, as well as non-opioid medications.<br>**Patient-reported** outcomes are evaluated using tools like the Brief Michigan Hand Questionnaire, Upper Extremity Score Mean, and the Patient-Reported Outcomes Measurement Information System (PROMIS) for Global Mental Health, Global Physical Health, Pain Interference Score, Pain Scale Score, and Quality of Life Score.<br>**Socioeconomic status** is assessed through self-reported education levels (ranging from less than high school to professional or graduate school) and family income brackets (e.g., less than $20,000 to more than $70,000). Additionally, the study includes variables for self-reported conditions such as anxiety, depression, COPD, coronary artery disease, hypertension, and others, providing a broad view of the patient’s overall health status and background. |
| **Postsurgical** | Grazal et al | 2022 | **Demographic, Preoperative and Postoperative.** The covariates used in the machine-learning models for this study included a wide range of demographic, clinical, military service, and geographic factors. Demographic covariates consisted of patient age, sex (male or female), race (White, Asian or Pacific Islander, Black, Western Hemisphere Indians, other, unknown, or missing), and marital status (divorced, married, single, widowed, etc.).<br>**Military-related factors** included military status (active duty, retired, dependents, etc.), rank group (cadet, enlisted, officer, warrant officer, or other), and sponsor’s military service branch (Army, Navy, Air Force, etc.). |

| | | | |
|---|---|---|---|
| | | | **Geographic variables** included the region of care (e.g., Alaska, North, West, South, and various international regions). Temporal variables encompassed the fiscal year (2012–2017).<br>**Clinical covariates** included opioid use patterns categorized into preoperative, perioperative, postoperative, and prolonged postoperative use. Additionally, physical comorbidities (e.g., cardiovascular disorders, renal failure, gastrointestinal disorders, osteoarthritis, asthma) and psychological comorbidities (e.g., PTSD, anxiety-related disorders, substance dependence) were included as covariates to assess their impact on the outcomes. |
| **Postsurgical** | Hur et al.,2021 | 2021 | **Demographic** variables such as age, gender, race, and education level were considered, with a diverse representation across different age groups (18–64 years), genders (predominantly female), and races (primarily White, followed by Hispanic and Black participants).<br>**Socioeconomic** status was captured through net worth categories ranging from under $25,000 to over $500,000. Clinical factors included the Charlson comorbidity score, history of tobacco use, and mental health disorders (e.g., mood disorders, anxiety, psychosis). The type of surgery (major or minor) was also analysed.<br>**Pain-related conditions** were detailed, encompassing a variety of pain disorders like arthritis, back problems, migraines, and other pain conditions.<br>**Preoperative factors**, such as the use of benzodiazepines and opioids within 30 days prior to surgery, were included to assess their impact on postoperative opioid use patterns. |
| **Postsurgical** | Karhade et al., a | 2019 | **Demographic** factors included age (median of 51 years), gender (52.6% female), race (10.6% non-White, 89.4% White), and ethnicity (2.1% Hispanic, 97.9% non-Hispanic). Marital status (64.1% married) and veteran status were also recorded. Patient disposition was classified into inpatient (16.2%) and outpatient (83.8%) categories, with preoperative deficits like myelopathy (25.4%) and radiculopathy (36.2%) being noted.<br>**Surgical factors** considered were multilevel surgery (45.5%) and history of previous spine surgery (4.7%).<br>**Preoperative** laboratory values, including haemoglobin, white blood cell count, platelet count, and creatinine levels, were analysed. Insurance status varied, with the majority having private insurance (74.1%), and smaller groups with Medicaid, Medicare, workers' compensation, or no insurance.<br>**Neighbourhood characteristics** such as median household income, median age, high school graduation rate, unemployment rate, and population density were included. Medication use prior to surgery was detailed, covering drugs like ACE inhibitors, ARBs, antidepressants, beta-blockers, benzodiazepines, gabapentin, NSAIDs, and antipsychotics. The duration of preoperative opioid use was categorized into none, less than 180 days, or more than 180 days.<br>**Comorbidities** like tobacco use (12.7%), alcohol abuse (2.4%), drug abuse (2.5%), diabetes (11.5%), renal failure (1.6%), depression (16.4%), psychoses (0.8%), and myocardial infarction (2.2%). Additional health conditions such as congestive heart failure, peripheral vascular disease, cerebrovascular accidents, hemiplegia/paraplegia, COPD, arrhythmias, valvular disease, liver disease, and malignancy were also considered. |
| **Postsurgical** | Karhade et al., b | 2019 | Demographic factors included age, sex, marital status (classified as married if legally married or in a common-law partnership), veteran status, race (categorized as White or non-White), and ethnicity (Hispanic or non-Hispanic).<br>**Procedural factors** encompassed the type of surgery performed, including fusion, surgical approach, multilevel surgery, and instrumentation, along with a history of previous spine surgeries.<br>**Laboratory values** were assessed, such as white blood cell count, haemoglobin levels, platelet count, creatinine, and prothrombin time (PT). Insurance status was categorized into Medicaid, workers' compensation, Medicare, or uninsured.<br>**Neighbourhood characteristics**, derived from U.S. Census Bureau American Community Survey data, included median household income, median age, high school graduation or General Equivalency Diploma (GED) attainment rate, unemployment rate, and population density.<br>**Preoperative medications** considered in the analysis included angiotensin-converting enzyme inhibitors, angiotensin receptor blockers, antidepressants, beta-2-agonists, beta-blockers, benzodiazepines, gabapentin, immunosuppressants, nonsteroidal anti-inflammatory drugs (NSAIDs), opioids, typical antipsychotics, and atypical antipsychotics.<br>**Preoperative comorbidities** recorded were tobacco use, drug abuse, diabetes, renal failure, malignancy, depression, psychoses, myocardial infarction, congestive heart failure, peripheral vascular disease, chronic obstructive pulmonary disease (COPD), arrhythmias, valvular disease, and liver disease.<br>The study also evaluated **preoperative opioid use duration**, categorized based on continuous prescriptions for more than 180 days before surgery, prescriptions for less than 180 days continuously, and no preoperative opioid prescriptions. These variables were included to assess their association with clinical outcomes following surgical procedures. |
| **Postsurgical** | Karhade et al., c | 2019 | **Demographic** variables comprised age, gender, race (categorized as White or Non-White), ethnicity (Hispanic or Non-Hispanic), marital status (classified as married or not married), and veteran status. Patient disposition was recorded as either inpatient or outpatient.<br>**Laboratory characteristics** included haemoglobin levels, white blood cell count, platelet count, and creatinine levels, which provide insights into the patient's baseline health status before surgery. Insurance status was categorized into Medicaid, Medicare, Free Care, or self-pay, reflecting different levels of healthcare access and financial coverage.<br>**Neighbourhood characteristics**, characteristics based on zip code data, such as median household income, median age, high school graduation rate, unemployment rate, and population density.<br>**Medication usage prior to surgery** was considered, including angiotensin-converting enzyme inhibitors, angiotensin receptor blockers, antidepressants, beta-2-agonists, beta-blockers, benzodiazepines, gabapentin, immunosuppressants, nonsteroidal anti-inflammatory drugs (NSAIDs), opioids, and antipsychotics.<br>**Preoperative comorbidities** evaluated included tobacco use, alcohol abuse, drug abuse, diabetes, renal failure, depression, psychosis, myocardial infarction, congestive heart failure, peripheral vascular disease, cerebrovascular accident, chronic obstructive pulmonary disease (COPD), arrhythmias, valvular disease, liver disease, and the presence of solid tumours.<br>**Preoperative opioid use** was categorized based on the duration of prescription use: no use, less than 180 days, more than 180 days, and sustained opioid prescription. This categorization helps to understand the potential risk associated with opioid use prior to surgery and its effect on postoperative outcomes. |

| | | | |
|---|---|---|---|
| **Postsurgical** | Karhade et al. | 2020 | **Demographic** predictors included age, sex, marital status, veteran status, race, and ethnicity. Patient disposition was categorized as either inpatient or outpatient. Operative diagnosis included specific conditions such as disk herniation, spondylolisthesis, and spinal stenosis.<br>**Procedural factors** considered were the type of surgery (fusion, approach, instrumented fusion), whether the intervention was multilevel, and any history of spine surgery within a year before the index procedure.<br>**Laboratory values** were also included as predictors, specifically white blood cell count, haemoglobin, platelet count, and creatinine levels, to evaluate the patient's preoperative physiological status. Insurance status was classified into Medicaid, Medicare, workers' compensation, uninsured, and private, reflecting the patient's access to healthcare and potential financial implications.<br>**Neighbourhood characteristics** were assessed using zip-code data, including median household income, median age, high school graduation or GED attainment rates, unemployment rate, and population density.<br>**Preoperative medications** were also evaluated, including angiotensin-converting enzyme (ACE) inhibitors, angiotensin II receptor blockers (ARBs), antidepressants, beta-2-agonists, beta-blockers, benzodiazepines, gabapentin, immunosuppressants, nonsteroidal anti-inflammatory drugs (NSAIDs), and antipsychotics.<br>**Preoperative comorbidities** considered in the study were tobacco use, alcohol abuse, drug abuse, diabetes, renal failure, depression, psychoses, myocardial infarction, congestive heart failure, peripheral vascular disease, cerebrovascular accident, chronic obstructive pulmonary disease (COPD), arrhythmias, valvular disease, liver disease, and malignancy. |
| **Postsurgical** | Katakam et al. | 2020 | **Demographics** (age, sex, race, ethnicity, marital status, veteran status), patient disposition (inpatient or outpatient status)<br>**Laboratory values** (haemoglobin, white blood cell count, platelet count, creatinine), insurance status (Medicaid, Medicare)<br>**Neighbourhood characteristics** (median household income, median age, high school education rate, unemployment rate).<br>**Preoperative medications** use was evaluated, including angiotensin-converting enzyme inhibitors, angiotensin II receptor blockers, antidepressants, beta-2-agonists, beta-blockers, benzodiazepines, gabapentin, immunosuppressants, nonsteroidal anti-inflammatory drugs (NSAIDs), opioids, and antipsychotics.<br>**Preoperative comorbidities** considered were tobacco use, alcohol abuse, drug abuse, diabetes, renal failure, depression, psychoses, myocardial infarction, congestive heart failure, peripheral vascular disease, cerebrovascular accident, chronic obstructive pulmonary disease (COPD), arrhythmias, valvular disease, liver disease, and malignancy. |
| **Postsurgical** | Klemt et al. | 2022 | **Demographics** (age, gender, body mass index [BMI], American Society of Anaesthesiologists [ASA] score, Charlson comorbidity index, insurance status, ethnicity, and marital status)<br>**Discharge disposition** (inpatient status)<br>**Preoperative comorbidities** (smoking, drinking, drug abuse, diabetes mellitus, psychosis, HIV, hepatitis, depression, renal failure, malignancy, vascular disease, myocardial infarction)<br>**Preoperative medications** (angiotensin-converting enzyme inhibitors, angiotensin receptor blockers, antidepressants, beta-2-agonists, benzodiazepines, immunosuppressants, opioids, antipsychotics).<br>Surgical variables included blood loss, spinal anaesthesia usage, tranexamic acid usage, and cemented component fixation. |
| **Postsurgical** | Kunze et al. | 2021 | **Demographics** (age, body mass index [BMI], female sex), race, and workers compensation status.<br>**Other characteristics** examined were drug allergies, sports participation, smoking status, presence of back pain, spine pathology, and whether anxiety or depression was reported. Additionally, symptom duration greater than two years, alpha angle on anterior-posterior (AP) radiograph, and Lateral Center Edge Angle (LCEA) were assessed.<br>**Pain levels** were measured using the Visual Analog Scale (VAS), and preoperative function was evaluated using the modified Harris Hip Score (mHHS), the Hip Outcome Score-Activities of Daily Living (HOS-ADL), and the Hip Outcome Score-Sport Specific Subscale (HOS-SS). The study also included data on opioid refills. No socioeconomic data. |
| **Postsurgical** | Lu et al. | 2022 | **Demographics** (age, female gender, body mass index [BMI]), preoperative pain levels, days of exercise, smoking status (current, former, never), duration of symptoms, and nonoperative treatments (physical therapy, injections or nerve blocks, medications, supplements, alternative treatments).<br>**Preoperative comorbidities** considered were arthritis, depression, high blood pressure, and thyroid problems. Preoperative patient-reported outcome measures (PROMs) included the International Knee Documentation Committee (IKDC) score, Knee Osteoarthritis Outcome Score (KOOS) for sport and activities, quality of life, physical symptoms, symptoms, pain, KOOS Junior (KOOS JR), activities of daily living, and the VR-12 Physical Component Summary (PCS) and Mental Component Summary (MCS) scores.<br>**Surgical procedures** included chondroplasty and meniscectomy, with preoperative opioid consumption and perioperative opioid medication usage also recorded. No socioeconomic data. |
| **Postsurgical** | Zhang et al. | 2020 | **Demographics** included age group, sex,<br>**Preoperative comorbidities** considered depression, chronic pulmonary disorder, alcohol and drug abuse, type of surgery (decompression with or without fusion), and preoperative opioid use.<br>**Postoperative variables** included opioid use within the first 30 days following surgery, as this period influences long-term opioid use (>180 days within a year).<br>**Opioid use** was analysed through various metrics: morphine milligram equivalents (MME) calculated using CDC dose conversions, daily opioid dose, changes in opioid dosage, average changes in MME, frequency of early refills, and high preoperative opioid use, defined as filling prescriptions with a duration ≥50% of the days between diagnosis and surgery. Prescriptions with excessively high MME or pill counts were excluded. No socioeconomic data. |
| **Persistent Opioid Use** | Bjarnadottir et al | 2022 | The dataset includes various variables related to military personnel, covering:<br>**Demographics:** Age, gender, race, ethnicity, marital status, education, and service details.<br>**Health and Medical History:** BMI, PULHES profile, opioid and psychotropic medication use, behavioural health diagnoses, pain levels, and cancer history.<br>**Medical Encounters:** Health encounters, pain and behavioural health visits, and case management.<br>**Opioid Use:** Total opioid prescriptions, days' supply, and categories of opioid potency. |

| | | | |
|---|---|---|---|
| | | | Variables are categorized into Demographic, deployment, physical fitness, healthcare utilisation and behaviour. Information about employment but could not find data on socioeconomic status. |
| **Persistent Opioid Use** | Calcaterra et al | 2018 | **Demographics** included gender, race, age at index admission, and insurance status.<br>**Presence of substance use disorders** such as tobacco, alcohol, stimulants, and opioids, as well as chronic and acute pain conditions.<br>**Mental health conditions**, including depression, anxiety, and bipolar disorder, were analysed, alongside chronic medical conditions like hypertension, respiratory disease, and diabetes. The Charlson Comorbidity Index was used to evaluate overall **comorbidity**.<br>**Discharge diagnoses,** surgical procedures performed during hospitalization, and the number of healthcare encounters in the year preceding the admission were also examined. Additionally, variables related to **opioid use**, such as opioid prescriptions, receipt of opioids at discharge, and morphine milligrams per hospital day, were included. The length of hospital stays and subsequent hospitalizations within 12 months post-discharge were also considered. No socioeconomic data. |
| **Persistent Opioid Use** | Held et al | 2023 | **Demographic** variables included age, categorized into groups such as under 50 years, 50 to 59, 60 to 69, 70 to 79, and 80 years and older, along with sex.<br>**Socioeconomic variables** assessed included the place of residence, distinguishing between German-speaking and non-German-speaking regions of Switzerland, insurance type (private or semiprivate versus standard), and the managed care model versus traditional insurance policies.<br>**Episode-specific variables** focused on initial opioid dosage, classified into ranges from less than 20 mg to 100 mg or more per day, as well as the number of opioid prescribers (single versus multiple) and previous opioid use, segmented into categories such as never used, more than two years ago, six months to two years ago, and within the last six months.<br>**Disease-specific risk factors** were evaluated using the Chronic Disease Score (CDS), which includes a range of chronic conditions like infections, inflammatory, renal, endocrine, cardiovascular, and psychiatric diseases.<br>**Comorbidities** were assessed as binary predictors.<br>**Comedication use** was analysed, with binary indicators for stimulants, bisphosphonates, muscle relaxants, nonopioid analgesics, and benzodiazepines. |
| **Persistent Opioid Use** | Johnson et al | 2022 | **Demographic** data included age and sex, though race and ethnicity were excluded due to inconsistent reporting across states.<br>**County-level socioeconomic data**, including income, unemployment, education, and urban-rural classification, were derived from patient ZIP codes and the Economic Research Service of the United States Department of Agriculture.<br>**Key features** assessed included prior prescriptions of gabapentin and pregabalin, daily morphine milligram equivalent (MME), opioid type, days' supply of prescriptions, and visit types (elective, urgent, emergent, trauma). |
| **Persistent Opioid Use** | Mohl et al | 2023 | **Demographic** variables encompass age at the index date, gender, ethnicity, race, and insurance type.<br>**Socioeconomic** factors are represented by ZIP code-based measures such as adjusted per capita income, percentage below 200% of poverty, median income, and rural/urban categorization.<br>**Clinical variables** include Body Mass Index (BMI), daily BMI change, and various diagnoses such as chronic pain, fibromyalgia, and specific types of osteoarthritis (e.g., hip, knee, shoulder).<br>**Patient-specific factors** involve mental health conditions like anxiety, depression, bipolar disorder, and substance use disorders including alcohol and drug dependence.<br>**Opioid-related variables** include the type and category of index opioid, opioid dependence, and opioid prescription counts.<br>**Medication use** is detailed through counts and unique counts of various drug classes, including benzodiazepines, anticonvulsants, antidepressants, NSAIDs, and tramadol.<br>**Additional health encounters** include emergency department visits and inpatient encounters, categorized by specialty such as orthopaedics, rheumatology, and pain management.<br>**Other variables** track prescribed treatments like corticosteroid injections and joint replacements, as well as health interventions such as prescribed exercise and manual therapy. |
| **Overdose** | Dong et al | 2021 | The manuscript states that the study extracted a total of 1,185 features, which include 414 diagnosis code features, 394 laboratory test features, 3 demographic features, 227 clinical events features, and 147 medication features. Although the manuscript references supplementary materials for the full list of predictors used in the analysis, the complete list could not be located within the supplementary materials. |
| **Overdose** | Dong et al | 2019 | Variables included **demographic, clinical events, medications, procedure, diagnosis**. Diagnosis codes, which detail diseases, symptoms, and poisonings. Procedure codes, indicating specific medical or surgical interventions, are included from both SPARCS and Health Facts datasets. Medication data, available only in the Health Facts dataset. Clinical events, such as symptoms and personal situations, are unique to the Health Facts dataset.<br>**Demographic** information, including age, gender, and race, is included in both datasets to improve predictions, while payment sources in SPARCS offer insights into patients' **socioeconomic** status. |
| **Overdose** | Gellad et al | 2023 | **Demographic** variables include identifiers such as age, gender, county, state, and postal code, as well as indicators for living in counties bordering neighbouring states.<br>**Prescription characteristics** cover various aspects of opioid use, including days since the last and first prescription, total morphine milligram equivalents (MME), the number and types of opioid prescriptions (e.g., oxycodone, acetaminophen/oxycodone), cumulative days of prescriptions, early refills, and overlapping prescriptions with benzodiazepines or carisoprodol.<br>**Other prescription data** include the number of benzodiazepine, buprenorphine, and stimulant prescriptions.<br>**Prescriber and pharmacy characteristics** encompass the number of opioid prescribers and pharmacies, with indicators for having more than five prescribers or pharmacies.<br>**Region-level variables** provide data on overdose and naloxone administration rates, including changes over time for emergency department visits, hospital admissions, and naloxone administrations, as well as rapid overdose deaths and overall opioid and heroin overdose rates. |
| **Overdose** | Lo-Ciganic et al, a | 2021 | **Socio-demographics, health status, prescription opioid use patterns, and human services and criminal justice records**. Text mentions a total of 290 predictors were included |

| | | | |
|---|---|---|---|
| **Overdose** | Lo-Ciganic et al, b | 2022 | **Sociodemographic, patient health status, use patterns of opioid and other non-opioid prescriptions, and provider-level and regional-level factors measured at baseline**. Text mentions a total of 284 predictors were included |
| **Overdose** | Lo-Ciganic et al, c | 2019 | **Sociodemographic** characteristics included age, sex, race/ethnicity, disability status as a criterion for Medicare eligibility, receipt of low-income subsidy, and urbanicity of the county of residence. Health status factors, such as the number of emergency department visits, were also considered.<br>**Opioid and medication use**, including: (1) total and mean daily morphine milligram equivalent (MME); (2) cumulative and continuous duration of opioid use, ensuring no gap longer than 32 days between prescriptions; (3) total number of opioid prescriptions overall and categorized by active ingredient; (4) type of opioid according to the US Drug Enforcement Administration's Controlled Substance Schedule (I-IV) and its duration of action; (5) number of opioid prescribers; (6) number of pharmacies filling opioid prescriptions; (7) frequency of early opioid prescription refills, defined as refilling more than 3 days before the previous prescription expires; (8) cumulative days of early prescription refills; (9) cumulative days of concurrent use of benzodiazepines and/or muscle relaxants; (10) number and duration of other relevant prescriptions, such as gabapentinoids; and (11) receipt of methadone hydrochloride or buprenorphine hydrochloride for opioid use disorder. |
| **Overdose** | Ripperger et al | 2021 | **Demographic** variables include age and sex.<br>**Prescription history** is characterized by the total number of prescriptions, distinct practitioners, pharmacies, and hospital identifiers.<br>**Additional variables** include the total morphine milligram equivalents (MME) prescribed, the distinction between short-acting and long-acting opioid prescriptions and overlapping prescriptions with benzodiazepines.<br>**Prior medications** for opioid use disorder and identifies opioid-naïve prescriptions as those without any opioid prescriptions in the past 45 days. Although race and ethnicity are not explicitly included in the models, the approach in this study involves modelling at the prescription level to enable detailed, time-dependent risk predictions, which are then aggregated at various levels including practice, pharmacy, local, county, and regional levels. |
| **Overdose** | Sun et al | 2020 | **Demographics, medical diagnoses, medication prescriptions, and healthcare utilization**. The analysis utilized seventy-eight candidate predictors selected based on subject matter expertise, encompassing various domains such as demographics, medical diagnoses, medication prescriptions, and healthcare utilization.<br>**Demographic** variables were recorded on the index date and modelled categorically, while medical diagnoses were represented as binary variables. Medication prescriptions and healthcare utilization were assessed as continuous variables.<br>**Medical diagnoses** were defined based on ICD-9-CM codes and evaluated over a 6-month period prior to each person-month of follow-up to ensure comprehensive capture of diagnoses.<br>**Medication prescriptions and healthcare utilization data** were assessed over the preceding 3-month period to reflect recent activity. |
| **Opioid Use Disorder** | Annis et al | 2022 | **Demographic** variables included age, sex, race, ethnicity, and marital status.<br>**Comorbidity levels** were measured by the Charlson Comorbidity Index, and indicators for alcohol use disorder, tobacco use disorder, mental illness, domestic violence, chronic pain, and previous hospitalizations were also included. Additionally, a previous diagnosis of opioid use disorder (OUD) was identified. Clinical conditions were evaluated over a 12-month baseline period using validated algorithms from the Centers for Medicare and Medicaid Chronic Conditions Warehouse. Psychiatric disorders were categorized to include anxiety disorders, bipolar disorder, depression, personality disorders, other psychotic disorders, post-traumatic stress disorder, and schizophrenia. Pain was categorized by conditions such as dorsopathies, migraine, fibromyalgia, and chronic pain. Physical accidents included transportation accidents, burns, corrosions, and injuries.<br>**Prescription** order data provided insights into prior use of analgesics, psychotropics, medications for OUD, and opioids. |
| **Opioid Use Disorder** | Banks et al | 2023 | **Demographic** variables included a scrambled social security number, gender, age, race (White), marital status, and whether the patient had and used VA insurance.<br>**Patient-condition variables** covered a variety of pre-existing conditions such as ADHD, adjustment reactions, alcohol use, amphetamine use, anxiety, arthritis, back pain, bipolar disorder, cannabis use, cerebrovascular disease, cocaine use, depression, hallucinogen use, musculoskeletal pain, neuropathy, obesity, other substance use, overweight, schizophrenia, smoking, PTSD, personality disorder, traumatic brain injury, and unspecified drug use. The target variable was opioid use disorder.<br>**Prescription-fill variables** included the total and maximum number of days early or late in picking up prescriptions, maximum morphine equivalent dosage, opioid use length in days, and the change in opioid dose from the beginning to the end of use. |
| **Opioid Use Disorder** | Dong et al | 2021 | It is mentioned that key variables included **diagnosis codes, procedure codes, laboratory tests, medications, clinical events, and demographic information,** and that 468 features were extracted, comprising 457 diagnosis features, 530 laboratory test features, 3 demographic features, 251 clinical event features, and 227 medication features. Laboratory test results, including numeric values and standardized interpretations (high, low, normal), were analysed for patterns and totals. Clinical events, encompassing symptoms, procedures, and personal information such as pain levels and smoking history, were included, reflecting a broad range of patient experiences.<br>**Demographic** variables like age, gender, and race or ethnicity were also included. Some information is provided but no specific detail of all included variables was found. |
| **Opioid Use Disorder** | Gao, W. et al | 2021 | **Demographic data** (age, gender, race, language, aid category)<br>**Disease indicators from medical and pharmacy claims**, and social determinants of health (SDOH) vulnerability based on self-reported issues like housing and food insecurity.<br>**Key opioid-related variables** included any opioid prescriptions, total pain medication prescriptions, high daily morphine milligram equivalents (MME), and classifications of short-acting versus long-acting opioids. Additionally, number of opioid prescribers and pharmacies, high-risk utilization indicators (such as mental health visits or admissions), and substance use disorders. |
| **Opioid Use Disorder** | Kashyap et al | 2023 | Characteristics of patient population are provided, but full list of predictors included in the analysis is not clearly stated. |

| | | | |
|---|---|---|---|
| **Opioid Use Disorder** | Liu et al | 2023 | **Demographic** variables like sex, age, family status, income level, and government aid.<br>**Key categories** included various substance use disorders such as opioid, sedative-hypnotic, amphetamine, cocaine, cannabis, and hallucinogen-related disorders. The study also looked at long-term opioid use, high and low opioid dosages, and chronic pain.<br>**Mental health conditions** were extensively covered, including disorders such as schizophrenia, bipolar disorder, depression, anxiety, and personality disorders.<br>**Additional variables** included the number of opioid prescribers, healthcare utilization metrics (inpatient, specialist, and emergency visits), and affiliations with primary care or focused clinics. The study also evaluated various psychosocial factors, including social income levels, family size, and psychological and behavioural factors. Full list is included in Supplementary Sections. |
| **Opioid Use Disorder** | Lo-Ciganic et al | 2020 | 269 potential predictors were included, covering **socio-demographics, health status, patterns of opioid use, and provider-level and regional- level factors in 3-month period**s, starting from three months before initiating opioids until development of OUD, loss of follow-up or end of 2016. Variables included patterns of opioid use, and patient, provider, and regional factors that were measured at baseline (i.e., within the three months before the first opioid fill) and in every 3-month period after prescription opioid initiation. |
| **Opioid Use Disorder** | Segal et al | 2020 | 436 predictor candidates, divided to six feature groups - **demographics, chronic conditions, diagnosis and procedures features, medication features, medical costs, and episode counts**. |
| **Opioid Dependency** | Che et al | 2017 | **Diagnosis, Procedures and Prescriptions**. No information on socioeconomic data. |
| **Opioid Dependency** | Ellis et al | 2019 | Information from electronic health records (EHR) including **lab tests, vital signs, medical procedures, prescriptions, and other data from millions of patients were used to predict opioid substance dependence.** Full list of variables included as predictors for reproducibility could not be located in the main text. |
| **Multiple Adverse Outcomes** | Fouladvand et al | 2023 | The study analysed a comprehensive set of variables, including all **medications, diagnoses, and procedures**, along with basic **demographic** data such as sex and age. Medications were categorized using Medispan generic product identifiers, diagnoses and procedures were grouped using Clinical Classification Software (CCS) codes. Initially, the dataset included 94 medication variables, 283 diagnosis variables, and 242 procedure variables, plus 2 demographic variables. To refine the dataset, features more than two standard deviations from the mean were excluded. The final set consisted of 269 variables: 50 medication variables, 138 diagnosis variables, 79 procedure variables, and 2 demographic variables (age and sex). No socioeconomic data was included. |
| **Multiple Adverse Outcomes** | Sharma et al, a | 2022 | **Demographic** information including age, sex, income, and rural/urban status.<br>**Drug utilization** by various Level 3 ATC codes that represent specific classes of medications, such as anaesthetics, antiepileptics, drugs for addictive disorders, anxiolytics, opioids, hypnotics and sedatives, cough suppressants, anti-inflammatory products, antihistamines, antibiotics, antidepressants, muscle relaxants, and others. The study also tracks the **dispensation of drugs** within the 30 days prior to opioid dispensation, which includes opioids like codeine, fentanyl, and hydrocodone, as well as benzodiazepines and Z-drugs such as alprazolam and zolpidem. Calculated fields in the analysis describe patterns of drug use, including the number of benzodiazepine/Z-drugs and opioids dispensed, unique drug molecules, prescribing physicians, and pharmacies involved. Additionally, it measures oral morphine equivalents consumed and instances of concurrent use of opioids with benzodiazepines or multiple opioids. |
| **Multiple Adverse Outcomes** | Sharma et al, b | 2021 | **Demographic** features such as age, sex, and income (assessed via Forward Sortation index from postal codes). It also included comorbidity history evaluated through ICD-based Elixhauser score categories and healthcare utilization metrics like the number of unique providers, hospital, and emergency department visits.<br>**Drug utilization** was analysed using Level 3 ATC codes, oral morphine equivalents, and metrics related to concurrent use of benzodiazepines, as well as the number of opioid and benzodiazepine dispensations and unique molecules. The data for these predictors spanned from 30 days to 5 years prior to opioid dispensation, allowing the models to capture both immediate and long-term risk factors. |
| **Multiple Adverse Outcomes** | Vunikili et al | 2021 | No clear information is provided on the full list of predictors included in the analysis. It is mentioned that a total of 25 features are chosen using data-driven techniques to represent the opioid prescription information of the selected cohort, but no clear information on demographics, or what variables were included. |
| **Mortality after Overdose** | Guo et al | 2021 | The study developed 348 predictors for their model, covering several main categories: **demographic characteristics, health status, prescription use patterns, details of opioid overdose events, and regional factors**.<br>**Demographic** data included age, sex, race, ethnicity, Medicaid eligibility reasons, urbanicity, and the calendar year of the opioid overdose event.<br>**Health status** variables encompassed diagnoses of opioid use disorder, other substance use disorders, comorbid conditions (such as HIV/AIDS and psychiatric disorders), and medical service utilization metrics like emergency department and inpatient visits. Information about the opioid overdose itself included specifics like the type of overdose, ambulance use, and severity.<br>**Prescription-related data** included metrics on opioid use such as total and mean daily morphine milligram equivalents and the cumulative and continuous duration of opioid prescriptions.<br>**Regional factors** were derived from publicly available databases linked via FIPS codes or zip codes and provided insights into healthcare resources, health status, and **socioeconomic** conditions. |

## Supplementary Table 3: Summary of Participants and Outcomes in Included Studies

| Category | Author | Year | Number participants | Number outcomes |
|---|---|---|---|---|
| **Seizure** | Behnoush et al | 2021 | 909 | 544 |
| **Postsurgical** | Anderson et al | 2020 | 10,919 | 1,373 |
| **Postsurgical** | Baxter et al | 2023 | 889 | 44 |
| **Postsurgical** | Gabriel et al | 2022 | 1,042 | 242 |
| **Postsurgical** | Giladi et al | 2023 | 1,656 | 312 |
| **Postsurgical** | Grazal et al | 2022 | 6,760 | 2,762 |
| **Postsurgical** | Hur et al.,2021 | 2021 | 112,898 | 13,377 |
| **Postsurgical** | Karhade et al., a | 2019 | 2,737 | 270 |
| **Postsurgical** | Karhade et al., b | 2019 | 5,413 | 416 |
| **Postsurgical** | Karhade et al., c | 2019 | 5,507 | 345 |
| **Postsurgical** | Karhade et al., d | 2020 | 8,435 | 359 |
| **Postsurgical** | Katakam et al. | 2020 | 12,542 | 1,125 |
| **Postsurgical** | Klemt et al. | 2022 | 8,873 | 643 |
| **Postsurgical** | Kunze et al. | 2021 | 775 | 141 |
| **Postsurgical** | Lu et al. | 2022 | 381 | 60 |
| **Postsurgical** | Zhang et al. | 2020 | 19,317 | 903 |
| **Persistent Opioid Use** | Bjarnadottir et al | 2022 | 209,774 | 1,547 |
| **Persistent Opioid Use** | Calcaterra et al | 2018 | 27,705 | 1,457 |
| **Persistent Opioid Use** | Held et al | 2023 | 418,564 | 71,863 |
| **Persistent Opioid Use** | Johnson et al | 2022 | 180,000 | 53,820 |
| **Persistent Opioid Use** | Mohl et al | 2023 | 33,894 | 2,925 |
| **Overdose** | Dong et al | 2021 | 7,284,389 | 60,646 |
| **Overdose** | Dong et al | 2019 | SPARCS Database (440,000)<br>Health Facts Database (110,000) | SPARCS Database (40,000)<br>Health Facts Database (10,000) |
| **Overdose** | Gellad et al | 2023 | 2,237,701 | 879 |
| **Overdose** | Lo-Ciganic et al | 2021 | 237,259 | 3,945 |
| **Overdose** | Lo-Ciganic et al | 2022 | 639,693 | 8,687 |
| **Overdose** | Lo-Ciganic et al | 2019 | 560,057 | 3,188 |
| **Overdose** | Ripperger et al | 2021 | 3,041,668 | 2,574 |
| **Overdose** | Sun et al | 2020 | 5,291,363 | 2,682 |
| **Opioid Use Disorder** | Annis et al | 2022 | 345,728 | 3,995 |
| **Opioid Use Disorder** | Banks et al | 2023 | 6,023,94 | 24,117 |
| **Opioid Use Disorder** | Dong et al | 2021 | 5,183,566 | 111,456 |
| **Opioid Use Disorder** | Gao, W. et al | 2021 | 2017 (n = 320,040)<br>2018 (n = 374,809)<br>2019 (n = 589,423) | Not specified |
| **Opioid Use Disorder** | Kashyap et al | 2023 | Unclear | Unclear |
| **Opioid Use Disorder** | Liu et al | 2023 | 873,955 | 12,860 |
| **Opioid Use Disorder** | Lo-Ciganic et al | 2020 | 361,527 | 1869 |
| **Opioid Use Disorder** | Segal et al | 2020 | 130,120 | 3239 |
| **Opioid Dependency** | Che et al | 2017 | 102,166 | 749 |

| | | | | |
|---|---|---|---|---|
| **Opioid Dependency** | Ellis et al | 2019 | 707,015 (controls) | 7,797 (Non-sparse Cases) |
| **Multiple Adverse Outcomes** | Fouladvand et al | 2023 | 96,974 | 10,464 |
| **Multiple Adverse Outcomes** | Sharma et al | 2022 | 853,324 | 14,916 |
| **Multiple Adverse Outcomes** | Sharma et al | 2021 | 392,979 | 6,608 |
| **Multiple Adverse Outcomes** | Vunikili et al | 2021 | 29,992 | 749 |
| **Mortality after Overdose** | Guo et al | 2021 | 9,686 | 207 |

## Supplementary Table 4: Internal and External Validation

| Author | Year | Internal Validation | External Validation |
|---|---|---|---|
| Behnoush et al | 2021 | Split by unspecified method | None |
| Anderson et al | 2020 | Cross-validation | None |
| Baxter et al | 2023 | Cross-validation | None |
| Gabriel et al | 2022 | Cross-validation | None |
| Giladi et al | 2023 | Yes | None |
| Grazal et al | 2022 | Cross-validation | None |
| Hur et al.,2021 | 2021 | Cross-validation | None |
| Karhade et al., a | 2019 | Cross-validation | None |
| Karhade et al., b | 2019 | Cross-validation | Yes. |
| Karhade et al., c | 2019 | Cross-validation | None |
| Karhade et al. | 2020 | Cross-validation | Yes. |
| Katakam et al. | 2020 | Cross-validation | None |
| Klemt et al. | 2022 | Cross-validation | None |
| Kunze et al. | 2021 | Cross-validation | None |
| Lu et al. | 2022 | Bootstrapping | None |
| Zhang et al. | 2020 | Cross-validation | None |
| Bjarnadottir et al | 2022 | Temporal split | None |
| Calcaterra et al | 2018 | Split by year | Yes. |
| Held et al | 2023 | Internal-external validation approach (repeatedly splitting the data set into a training and a validation set of sizes 90% and 10%) | No |
| Johnson et al | 2022 | Cross-validation | None |
| Mohl et al | 2023 | Cross-validation | None |
| Dong et al | 2021 | Random split | No |
| Dong et al | 2019 | Split by Gini impurity | No |
| Gellad et al | 2023 | Cross-validation | No |
| Lo-Ciganic et al | 2021 | Random split | No |

| | | | |
|---|---|---|---|
| Lo-Ciganic et al | 2022 | Randomly and equally divided into three datasets: training, testing, and internal validation. | Yes. |
| Lo-Ciganic et al | 2019 | Random split | No |
| Ripperger et al | 2021 | Random split | No |
| Sun et al | 2020 | Random split | No |
| Annis et al | 2022 | Cross-validation | None |
| Banks et al | 2023 | Cross-validation | No |
| Dong et al | 2021 | Unclear | No |
| Gao, W. et al | 2021 | Split by year | None |
| Kashyap et al | 2023 | | No |
| Liu et al | 2023 | Cross-validation | None |
| Lo-Ciganic et al | 2020 | Split by population and class prevalence | No |
| Segal et al | 2020 | Random Assignment | No |
| Che et al | 2017 | Cross-validation | No |
| Ellis et al | 2019 | Cross-validation | No |
| Fouladvand et al | 2023 | Cross-validation | Yes |
| Sharma et al | 2022 | Cross-validation | No |
| Sharma et al | 2021 | Yes | No |
| Vunikili et al | 2021 | Cross-validation | No |
| Guo et al | 2021 | Cross-validation | No |

**Supplementary Table 5: Calibration reported by the studies**

| Author | Year | Calibration Measures Reported | Value | Model |
|---|---|---|---|---|
| **Guo et al** | 2021 | Evaluated using calibration plots and Brier Score | Calibration curves provided without detailed results of intercept and slope | Not provided |
| **Fouladvand et al** | 2023 | Not performed | Not performed | Not performed |
| **Sharma et al** | 2022 | Evaluated using calibration plot and Brier Score | Brier Score: 0.0236 (No confidence intervals reported) | XGBoost |
| **Sharma et al** | 2021 | Evaluated using calibration plots | Calibration curves provided without detailed results of intercept and slope | Not provided |
| **Vunikili et al** | 2021 | Not performed | NA | NA |
| **Che et al** | 2017 | Not performed | NA | NA |
| **Ellis et al** | 2019 | Not performed | NA | NA |
| **Kashyap et al** | 2023 | Evaluated using calibration plots and Brier Score | 0.146 (No confidence intervals reported) | Deep Learning |

| | | | | |
|---|---|---|---|---|
| **Lo-Ciganic et al** | 2020 | Performed according to the text but further details not provided | | Not provided |
| **Annis et al** | 2022 | Not performed | NA | NA |
| **Banks et al** | 2023 | Not performed | NA | NA |
| **Gao, W. et al** | 2021 | Not performed | NA | NA |
| **Liu et al** | 2023 | Not performed | NA | NA |
| **Segal et al** | 2020 | Not performed | NA | NA |
| **Dong et al** | 2021 | Not performed | NA | NA |
| **Gellad et al** | 2023 | Evaluated using calibration plots and Brier Score | 0.0004 (No confidence intervals reported) | Gradient Boosting Machine |
| **Lo-Ciganic et al** | 2021 | Performed according to the text but further details not provided | | Not provided |
| **Lo-Ciganic et al** | 2022 | Evaluated using calibration plots | Calibration curves provided without detailed results of intercept and slope | Not provided |
| **Lo-Ciganic et al** | 2019 | Evaluated using calibration plots | Performed but further details not provided | Not provided |
| **Ripperger et al** | 2021 | Evaluated using calibration plots and Brier Score | 0.0001305 (No confidence intervals reported) | Ensemble model |
| **Sun et al** | 2020 | Evaluated using calibration plots and Brier Score | 0.00002662 (No confidence intervals reported) | Elastic net model |
| **Dong et al** | 2021 | Not performed | NA | NA |
| **Dong et al** | 2019 | Not performed | NA | NA |
| **Held et al** | 2023 | Scaled Brier Score | 48.5% | GLM |
| **Johnson et al** | 2022 | Evaluated using calibration curves in supplementary file | Calibration curves provided without detailed results of intercept and slope | Not provided |
| **Mohl et al** | 2023 | Evaluated using calibration plots | Calibration intercept: 0 Calibration Slope 0.97 | Elastic-Net Penalized Logistic Regression |
| **Bjarnadottir et al** | 2022 | Not performed | NA | NA |
| **Calcaterra et al** | 2018 | Not performed | NA | NA |
| **Anderson et al** | 2020 | Evaluated using calibration plots and Brier Score | Brier Score = 0.10 (CI 0.09 - 0.11) | Gradient Boosting Machine |
| **Giladi et al** | 2023 | Evaluated using calibration plots and Brier Score | Brier Score = 0.136 (No confidence intervals reported) | XGBoost |
| **Grazal et al** | 2022 | Evaluated using calibration plots and Brier Score | Brier Score = 0.21 (95% CI 0.20-0.22) | Artificial Neural Network |

| | | | | |
|---|---|---|---|---|
| **Karhade et al., a** | 2019 | Evaluated using calibration plots and Brier Score | Brier Score = 0.076 (CI 0.072-0.078) | Stochastic Gradient Boosting Algorithm |
| **Karhade et al., b** | 2019 | Evaluated using calibration plots and Brier Score | Brier Score = 0.065 (No confidence intervals reported) | Stochastic Gradient Boosting Algorithm |
| **Karhade et al., c** | 2019 | Evaluated using calibration plots and Brier Score | Brier Score = Ranged between models from 0.052 to 0.056 | Elastic-Net Penalized Logistic Regression |
| **Karhade et al.** | 2020 | Evaluated using calibration plots and Brier Score | Brier Score = 0.039 (No confidence intervals reported) | Elastic-Net Penalized Logistic Regression |
| **Katakam et al.** | 2020 | Evaluated using calibration plots and Brier Score | Brier Score = 0.073 (No confidence intervals reported) | Elastic-Net Penalized Logistic Regression |
| **Klemt et al.** | 2022 | Evaluated using calibration plots and Brier Score | Brier Score = 0.037 (No confidence intervals reported) | Neural Network |
| **Kunze et al.** | 2021 | Evaluated using calibration plots and Brier Score | Brier Score = 0.13 (CI 0.093 - 0.16) | Elastic-Net Penalized Logistic Regression |
| **Lu et al.** | 2022 | Evaluated using calibration plots and Brier Score | Brier Score = 0.12 (CI 0.10–0.15) | Support Vector Machine |
| **Zhang et al.** | 2020 | Evaluated using calibration plots and Brier Score | Brier Score = 0.39 (No confidence intervals reported) | Stochastic Gradient Boosting Algorithm |
| **Baxter et al** | 2023 | Not performed | NA | NA |
| **Gabriel et al** | 2022 | Not performed | NA | NA |
| **Hur et al.,2021** | 2021 | Not performed | NA | NA |
| **Behnoush et al** | 2021 | Not performed | NA | NA |

**Supplementary Table 6: Keywords and Boolean operators to construct search strategy**

**OVID Medline** (R)- 411 studies

| | |
|---|---|
| 1 | Opioid.mp. or Analgesics, Opioid/ |
| 2 | Opiate.mp. or Opiate Alkaloids/ |
| 3 | codeine.mp. or Codeine/ |
| 4 | dihydrocodeine.mp. |
| 5 | meptazinol.mp. or Meptazinol/ |
| 6 | tramadol.mp. or Tramadol/ |
| 7 | tapentadol.mp. or Tapentadol/ |

| | |
|---|---|
| 8 | morphine.mp. or Morphine/ |
| 9 | oxycodone.mp. or Oxycodone/ |
| 10 | fentanyl.mp. or Fentanyl/ |
| 11 | Buprenorphine/ or buprenorphine.mp. |
| 12 | diamorphine.mp. or Heroin/ |
| 13 | hydromorphone.mp. or Hydromorphone/ |
| 14 | hydrocodone.mp. or Hydrocodone/ |
| 15 | pethidine.mp. or Meperidine/ |
| 16 | Diagnosis, Computer-Assisted/ or Machine Learning.mp. or Artificial Intelligence/ or Machine Learning/ or Algorithms/ or Pattern Recognition, Automated/ |
| 17 | 1 and 16 |
| 18 | Opiate Overdose/ or Drug Overdose/ or overdose.mp. |
| 19 | 17 and 18 |
| 20 | Models, Statistical/ or model selection.mp. |
| 21 | model prediction.mp. |
| 22 | LASSO.mp. |
| 23 | Random Forest.mp. |
| 24 | Regression Analysis/ or regression.mp. |
| 25 | gradient boosting machine.mp. |
| 26 | Neural Networks, Computer/ or neural network.mp. |
| 27 | k-means clustering.mp. or Cluster Analysis/ |
| 28 | naive bayes.mp. |
| 29 | Support vector machine.mp. or Support Vector Machine/ |
| 30 | back propagation neural network.mp. or Pattern Recognition, Automated/ |
| 31 | BPNN.mp. |
| 32 | boruta.mp. |
| 33 | feature selection.mp. |
| 34 | XGBoost.mp. |
| 35 | hierarchical clustering.mp. |
| 36 | Linear bayes normal classifier.mp. |
| 37 | deep learning.mp. or Deep Learning/ |
| 38 | 1 or 2 or 3 or 4 or 5 or 6 or 7 or 8 or 9 or 10 or 11 or 12 or 13 or 14 or 15 |
| 39 | 16 or 20 or 21 or 22 or 23 or 24 or 25 or 26 or 27 or 28 or 29 or 30 or 31 or 32 or 33 or 34 or 35 or 36 or 37 |
| 40 | 38 and 39 |

| | |
|---|---|
| 41 | 18 and 40 |
| 42 | "Persistent use".mp. |
| 43 | "High MME".mp. |
| 44 | high morphine milligram equivalents.mp. |
| 45 | addiction.mp. or Addiction Medicine/ |
| 46 | overdose.mp. |
| 47 | Mortality/ |
| 48 | "postoperative opioid use".mp. |
| 49 | "use disorder".mp. |
| 50 | "prescription".mp. or Prescriptions/ |
| 51 | "high risk".mp. |
| 52 | "long-term use".mp. |
| 53 | "high dose opioids".mp. |
| 54 | dependence.mp. |
| 55 | Death/ |
| 56 | ("adverse outcomes" or "adverse events").mp. [mp=title, book title, abstract, original title, name of substance word, subject heading word, floating sub-heading word, keyword heading word, organism supplementary concept word, protocol supplementary concept word, rare disease supplementary concept word, unique identifier, synonyms, population supplementary concept word, anatomy supplementary concept word] |
| 57 | Hospitalisation.mp. [mp=title, book title, abstract, original title, name of substance word, subject heading word, floating sub-heading word, keyword heading word, organism supplementary concept word, protocol supplementary concept word, rare disease supplementary concept word, unique identifier, synonyms, population supplementary concept word, anatomy supplementary concept word] |
| 58 | Recovery.mp. [mp=title, book title, abstract, original title, name of substance word, subject heading word, floating sub-heading word, keyword heading word, organism supplementary concept word, protocol supplementary concept word, rare disease supplementary concept word, unique identifier, synonyms, population supplementary concept word, anatomy supplementary concept word] |
| 59 | Hospitalization.mp. [mp=title, book title, abstract, original title, name of substance word, subject heading word, floating sub-heading word, keyword heading word, organism supplementary concept word, protocol supplementary concept word, rare disease supplementary concept word, unique identifier, synonyms, population supplementary concept word, anatomy supplementary concept word] |
| 60 | emergency.mp. [mp=title, book title, abstract, original title, name of substance word, subject heading word, floating sub-heading word, keyword heading word, organism supplementary concept word, protocol supplementary concept word, rare disease supplementary concept word, unique identifier, synonyms, population supplementary concept word, anatomy supplementary concept word] |
| 61 | dose reduction.mp. or Drug Tapering/ |
| 62 | "drug management".mp. |
| 63 | "substance use disorder".mp. or Substance-Related Disorders/ |

| | |
|---|---|
| 64 | "risk factors".mp. or Risk Factors/ |
| 65 | "opioid-related".mp. |
| 66 | Opioid-Related Disorders/ |
| 67 | orod.mp. |
| 68 | 42 or 43 or 44 or 45 or 46 or 47 or 48 or 49 or 50 or 51 or 52 or 53 or 54 or 55 or 56 or 61 or 62 or 63 or 64 or 65 or 66 or 67 |
| 69 | 38 and 39 and 68 |
| 70 | 1 or 2 or 3 or 4 or 5 or 6 or 7 or 8 or 9 or 10 or 11 or 12 or 13 or 14 or 15 |
| 71 | 16 or 20 or 21 or 22 or 23 or 24 or 25 or 26 or 27 or 28 or 29 or 30 or 31 or 32 or 33 or 34 or 35 or 36 or 37 |
| 72 | 70 and 71 |
| 73 | 18 or 42 or 43 or 44 or 45 or 46 or 47 or 48 or 49 or 50 or 51 or 52 or 53 or 54 or 55 or 56 or 57 or 58 or 59 or 60 or 61 or 62 or 63 or 64 or 65 or 66 or 67 |
| 74 | 70 and 71 and 73 |
| 75 | 16 or 21 or 23 or 25 or 26 or 27 or 28 or 29 or 30 or 31 or 32 or 33 or 34 or 35 or 36 or 37 |
| 76 | 70 and 73 and 75 |
| 77 | machine learning.mp. or Artificial Intelligence/ or Machine Learning/ |
| 78 | 21 or 22 or 23 or 25 or 26 or 27 or 28 or 29 or 30 or 31 or 32 or 33 or 34 or 35 or 36 or 37 or 77 |
| 79 | 70 and 73 and 78 |

**PubMed** (498 results)

(( (opioid) OR (opiate) OR (codeine) OR (dihydrocodeine) OR (meptazinol) OR (tramadol) OR (tapentadol) OR (morphine) OR (oxycodone) OR (fentanyl) OR (buprenorphine) OR (diamorphine) OR (hydromorphone) OR (hydrocodone) OR (pethidine) ) AND ( (machine learning) OR (machine-learning) OR (artificial intelligence) OR (model prediction) OR lasso OR(random forest) OR (gradient boosting machine) OR (neural network) OR (k-means clustering) OR (naive bayes) OR (support vector machine) OR svms OR (back propagation neural network) OR bpnn OR boruta OR (feature selection) OR xgboost OR (hierarchical clustering) OR (linear bayes normal classifier) OR (deep learning) ) AND ( dependence OR (persistent use) OR (high MME) OR (high morphine milligram equivalents) OR addiction OR overdose OR mortality OR (postoperative opioid use) OR (use disorder) OR prescription OR (high risk) OR (long-term use) OR (high dose) OR (high-dose) OR dependence OR death OR (adverse outcomes) OR (dose reduction) OR (drug management) OR (substance use disorder) OR (risk factors) OR (opioid related disorders) OR orod OR (hospitalization) OR (hospitalisation) OR (emergency) OR (recovery) )) AND (prescription)

SCOPUS (406 documents)

( TITLE-ABS-KEY ( prescription ) AND TITLE-ABS-KEY ( opioid OR opiate OR codeine OR dihydrocodeine OR meptazinol OR tramadol OR tapentadol OR morphine OR oxycodone OR fentanyl OR buprenorphine OR diamorphine OR hydromorphone OR hydrocodone OR pethidine ) AND TITLE-ABS-KEY ( ( machine AND learning ) OR machine-learning OR ( artificial AND intelligence ) OR ( model AND prediction ) OR lasso OR ( random AND forest ) OR ( gradient AND boosting AND machine ) OR ( neural AND network ) OR ( k-means AND clustering ) OR ( naive AND bayes ) OR ( support AND vector AND machine ) OR svms OR ( back AND propagation AND neural AND network ) OR bpnn OR boruta OR ( feature AND selection ) OR xgboost OR ( hierarchical AND clustering ) OR ( linear AND bayes AND normal AND classifier ) OR ( deep AND learning ) ) AND TITLE-ABS-KEY ( dependence OR ( persistent AND use ) OR ( high AND mme ) OR ( high AND morphine AND milligram AND equivalents ) OR addiction OR overdose OR mortality OR ( postoperative AND opioid AND use ) OR ( use AND disorder ) OR prescription OR ( high AND risk ) OR ( long-term AND use ) OR dependence OR death OR ( adverse AND outcomes ) OR ( dose AND reduction ) OR ( substance AND use AND disorder ) OR ( risk AND factors ) OR ( opioid AND related AND disorders ) OR ( hospitalization ) OR ( hospitalisation ) OR ( emergency ) ) )

**Supplementary Figure 1. Summary of Risk of Bias Assessment for Machine Learning Algorithms Predicting Postoperative Opioid Use**

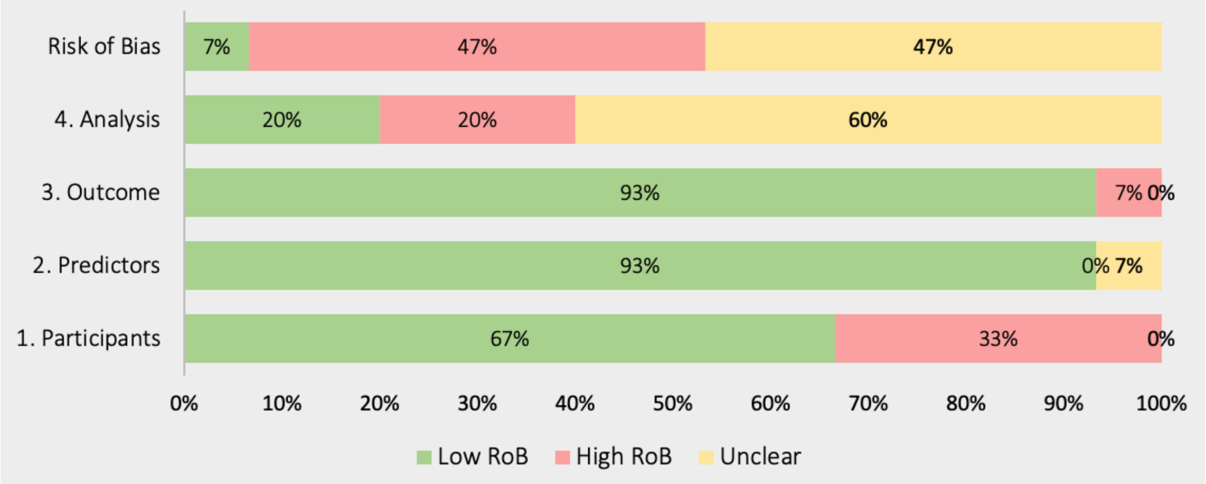


*The above plots present the risk of bias assessment for the 15 studies identified that developed prognostic clinical prediction models. Of these, only one study provided sufficient transparent reporting to be classified as having a low risk of bias, based on the PROBAST assessment tool.*

**Supplementary Figure 2. Summary of Risk of Bias assessment for Machine Learning Algorithms that Predict Opioid Use Disorder**

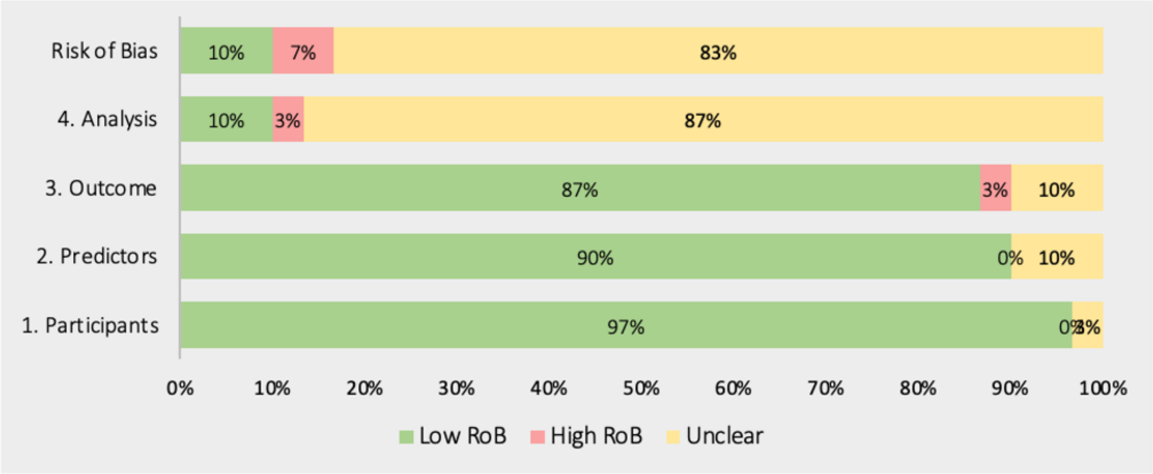


*The above plots present the risk of bias assessment for the eight studies identified that developed prognostic clinical prediction models. Of these, three studies provided sufficient transparent reporting to be classified as having a low risk of bias, based on the PROBAST assessment tool.*

**Supplementary Figure 3. Summary of Risk of Bias assessment for Machine Learning Algorithms that Predict Opioid Overdose**

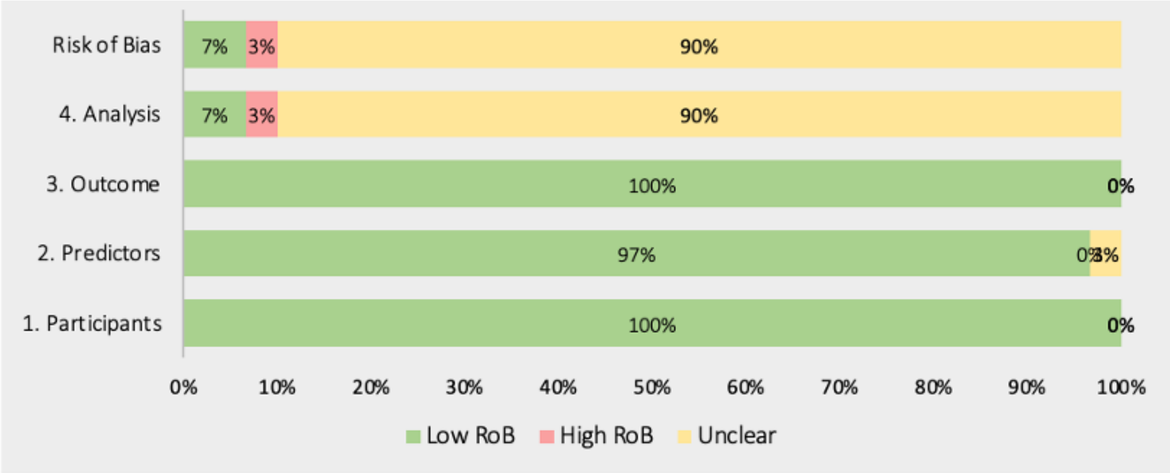


*The above plots present the risk of bias assessment for the eight studies identified that developed prognostic clinical prediction models to predict opioid overdose. Of these, two studies provided sufficient transparent reporting to be classified as having a low risk of bias, based on the PROBAST assessment tool.*

**Supplementary Figure 4. Summary of Risk of Bias assessment for Machine Learning Algorithms that Predict diverse opioid-related harms**

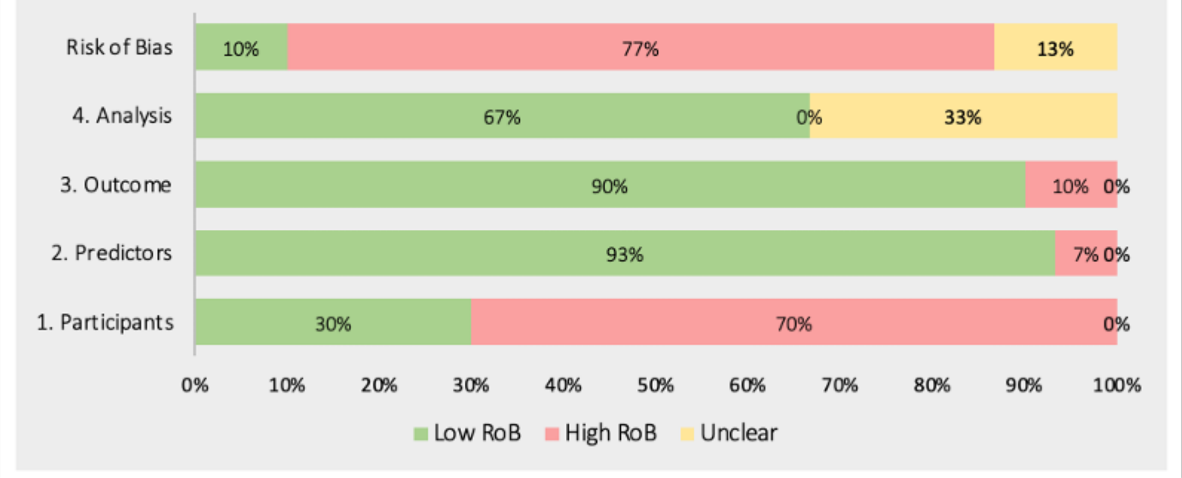


*The above plots present the risk of bias assessment for the four studies identified that developed prognostic clinical prediction models to predict multiple diverse outcomes.*

PRISMA 2020 Checklist

| Section and Topic | Item # | Checklist item | Location where item is reported |
|---|---|---|---|
| **TITLE** | | | |
| Title | 1 | Identify the report as a systematic review. | 1 |
| **ABSTRACT** | | | |
| Abstract | 2 | See the PRISMA 2020 for Abstracts checklist. | 2 |
| **INTRODUCTION** | | | |
| Rationale | 3 | Describe the rationale for the review in the context of existing knowledge. | 3 |
| Objectives | 4 | Provide an explicit statement of the objective(s) or question(s) the review addresses. | 4 |
| **METHODS** | | | |
| Eligibility criteria | 5 | Specify the inclusion and exclusion criteria for the review and how studies were grouped for the syntheses. | 4 & 5 |
| Information sources | 6 | Specify all databases, registers, websites, organisations, reference lists and other sources searched or consulted to identify studies. Specify the date when each source was last searched or consulted. | 4 |
| Search strategy | 7 | Present the full search strategies for all databases, registers and websites, including any filters and limits used. | Supplementary Section |
| Selection process | 8 | Specify the methods used to decide whether a study met the inclusion criteria of the review, including how many reviewers screened each record and each report retrieved, whether they worked independently, and if applicable, details of automation tools used in the process. | 6 & 7 |
| Data collection process | 9 | Specify the methods used to collect data from reports, including how many reviewers collected data from each report, whether they worked independently, any processes for obtaining or confirming data from study investigators, and if applicable, details of automation tools used in the process. | 6 |
| Data items | 10a | List and define all outcomes for which data were sought. Specify whether all results that were compatible with each outcome domain in each study were sought (e.g. for all measures, time points, analyses), and if not, the methods used to decide which results to collect. | 8 |
| | 10b | List and define all other variables for which data were sought (e.g. participant and intervention characteristics, funding sources). Describe any assumptions made about any missing or unclear information. | Table 3 & Supplementary Section |
| Study risk of bias assessment | 11 | Specify the methods used to assess risk of bias in the included studies, including details of the tool(s) used, how many reviewers assessed each study and whether they worked independently, and if applicable, details of automation tools used in the process. | 6 |
| Effect measures | 12 | Specify for each outcome the effect measure(s) (e.g. risk ratio, mean difference) used in the synthesis or presentation of results. | 7 |
| Synthesis methods | 13a | Describe the processes used to decide which studies were eligible for each synthesis (e.g. tabulating the study intervention characteristics and comparing against the planned groups for each synthesis (item #5)). | Supplementary Section |
| | 13b | Describe any methods required to prepare the data for presentation or synthesis, such as handling of missing summary statistics, or data conversions. | 6 |
| | 13c | Describe any methods used to tabulate or visually display results of individual studies and syntheses. | 6 |
| | 13d | Describe any methods used to synthesize results and provide a rationale for the choice(s). If meta-analysis was performed, describe the model(s), method(s) to identify the presence and extent of statistical heterogeneity, and software package(s) used. | 6 |
| | 13e | Describe any methods used to explore possible causes of heterogeneity among study results (e.g. subgroup analysis, meta-regression). | 6 |
| | 13f | Describe any sensitivity analyses conducted to assess robustness of the synthesized results. | NA |
| Reporting bias | 14 | Describe any methods used to assess risk of bias due to missing results in a synthesis (arising from reporting biases). | 6 |

| Section and Topic | Item # | Checklist item | Location where item is reported |
|---|---|---|---|
| assessment | | | |
| Certainty assessment | 15 | Describe any methods used to assess certainty (or confidence) in the body of evidence for an outcome. | NA |
| **RESULTS** | | | |
| Study selection | 16a | Describe the results of the search and selection process, from the number of records identified in the search to the number of studies included in the review, ideally using a flow diagram. | 8 |
| | 16b | Cite studies that might appear to meet the inclusion criteria, but which were excluded, and explain why they were excluded. | Figure 1 |
| Study characteristics | 17 | Cite each included study and present its characteristics. | Table 3 & Supplementary Section |
| Risk of bias in studies | 18 | Present assessments of risk of bias for each included study. | Table 2 |
| Results of individual studies | 19 | For all outcomes, present, for each study: (a) summary statistics for each group (where appropriate) and (b) an effect estimate and its precision (e.g. confidence/credible interval), ideally using structured tables or plots. | 8 – 15 |
| Results of syntheses | 20a | For each synthesis, briefly summarise the characteristics and risk of bias among contributing studies. | 8 – 15 |
| | 20b | Present results of all statistical syntheses conducted. If meta-analysis was done, present for each the summary estimate and its precision (e.g. confidence/credible interval) and measures of statistical heterogeneity. If comparing groups, describe the direction of the effect. | Table 3 |
| | 20c | Present results of all investigations of possible causes of heterogeneity among study results. | 8 – 15 |
| | 20d | Present results of all sensitivity analyses conducted to assess the robustness of the synthesized results. | NA |
| Reporting biases | 21 | Present assessments of risk of bias due to missing results (arising from reporting biases) for each synthesis assessed. | Table 2 & Supplementary Section |
| Certainty of evidence | 22 | Present assessments of certainty (or confidence) in the body of evidence for each outcome assessed. | NA |
| **DISCUSSION** | | | |
| Discussion | 23a | Provide a general interpretation of the results in the context of other evidence. | 16 |
| | 23b | Discuss any limitations of the evidence included in the review. | 19 |
| | 23c | Discuss any limitations of the review processes used. | 19 |
| | 23d | Discuss implications of the results for practice, policy, and future research. | 17 - 19 |
| **OTHER INFORMATION** | | | |
| Registration and protocol | 24a | Provide registration information for the review, including register name and registration number, or state that the review was not registered. | Review was not registered |
| | 24b | Indicate where the review protocol can be accessed, or state that a protocol was not prepared. | NA |
| | 24c | Describe and explain any amendments to information provided at registration or in the protocol. | NA |
| Support | 25 | Describe sources of financial or non-financial support for the review, and the role of the funders or sponsors in the review. | 1 |
| Competing interests | 26 | Declare any competing interests of review authors. | 20 |

# PRISMA 2020 Checklist

| Section and Topic | Item # | Checklist item | Location where item is reported |
|---|---|---|---|
| Availability of data, code and other materials | 27 | Report which of the following are publicly available and where they can be found: template data collection forms; data extracted from included studies; data used for all analyses; analytic code; any other materials used in the review. | Supplementary Section |

*From:* Page MJ, McKenzie JE, Bossuyt PM, Boutron I, Hoffmann TC, Mulrow CD, et al. The PRISMA 2020 statement: an updated guideline for reporting systematic reviews. BMJ 2021;372:n71. doi: 10.1136/bmj.n71

For more information, visit: http://www.prisma-statement.org/
